# Quantitative analysis of mutation and selection pressures on base composition skews in bacterial chromosomes

**DOI:** 10.1186/1471-2164-8-286

**Published:** 2007-08-21

**Authors:** Chi Chen, Carton W Chen

**Affiliations:** 1 Institute of Biomedical Informatics, National Yang-Ming University, Shih-Pai, Taipei 111, Taiwan; 2 Department of Life Sciences and Institute of Genome Sciences, National Yang-Ming University, Shih-Pai, Taipei 111, Taiwan

## Abstract

**Background:**

Most bacterial chromosomes exhibit asymmetry of base composition with respect to leading *vs*. lagging strands (GC and AT skews). These skews reflect mainly those in protein coding sequences, which are driven by asymmetric mutation pressures during replication and transcription (notably asymmetric cytosine deamination) plus subsequent selection for preferred structures, signals, amino acid or codons. The transcription-associated effects but not the replication-associated effects contribute to the overall skews through the uneven distribution of the coding sequences on the leading and lagging strands.

**Results:**

Analysis of 185 representative bacterial chromosomes showed diverse and characteristic patterns of skews among different clades. The base composition skews in the coding sequences were used to derive quantitatively the effect of replication-driven mutation plus subsequent selection ('replication-associated pressure', RAP), and the effect of transcription-driven mutation plus subsequent selection at translation level ('transcription-associate pressure', TAP). While different clades exhibit distinct patterns of RAP and TAP, RAP is absent or nearly absent in some bacteria, but TAP is present in all. The selection pressure at the translation level is evident in all bacteria based on the analysis of the skews at the three codon positions. Contribution of asymmetric cytosine deamination was found to be weak to TAP in most phyla, and strong to RAP in all the Proteobacteria but weak in most of the Firmicutes. This possibly reflects the differences in their chromosomal replication machineries. A strong negative correlation between TAP and G+C content and between TAP and chromosomal size were also revealed.

**Conclusion:**

The study reveals the diverse mutation and selection forces associated with replication and transcription in various groups of bacteria that shape the distinct patterns of base composition skews in the chromosomes during evolution. Some closely relative species with distinct base composition parameters are uncovered in this study, which also provides opportunities for comparative bioinformatic and genetic investigations to uncover the underlying principles for mutation and selection.

## Background

A genome contains coding information that specifies protein and RNA sequences and structural information that specifies local DNA conformation involved in interactions with proteins. On top of these is the subtle global tendency of a genome to move toward a preferred nucleotide composition and distribution that are characteristic for each clade. Most notable is the G+C content, which vary widely (between 25% to 72%) among the prokaryotes. The preferred G+C content is conserved among closely related species, as are the relative abundance of dinucleotides, trinucleotides, and tetranucleotides [for review, [1-4]].

In addition, in most bacterial chromosomes, mononucleotides exhibit a biased distribution between the two replicating (leading *vs*. lagging) strands. GC skew, as expressed by (*G*-*C*)/(*G*+*C*), and AT skew, expressed by (*A*-*T*)/(*A*+*T*), of bacterial chromosomes were first noticed by Lobry [5] in *Escherichia coli, Bacillus subtilis*, and *Haemophillus influenzae*, and later by Mrazek and Karlin [6] in *Mycoplasma genitalium, Mycoplasma pneumoniae*, and *Helicobacter pylori*. It was noticed that GC skews (and, to lesser extent, AT skews) exhibit a striking sign switch at the replication origin (*oriC*) and another one at the termination region in many bacterial chromosomes. From an analysis of a limited number (9 to 36) of bacterial chromosomes, it has been proposed that there is an overall excess of purines ('purine excess') or keto bases G and T ('keto excess') in the protein coding sequences (CDS) [7-9]. These base composition skews have been recently reviewed [10-12].

The composition skew may be extended to include a number of oligomer sequences, which are known to be or are likely to be implicated in replication, recombination, and/or repair process of genomes [13,14]. A classical example is the octameric Chi sequence (CGTGGTGG) in *E. coli*, which serves as a signal for recombinational repair of double strand breaks, and is important to the rescuing of broken replication forks [[15] for a concise review]. Another example is the Rag motif (RGNAGGGS) in the *E. coli *chromosome, the skew of which shift abruptly at the terminus of replication [13,16]. Chi and Rag motifs together account for about 7% of the global GC skew of the *E. coli *chromosome [14].

Base composition skews are shaped by asymmetric accumulation of specific mutations, which are determined at two levels, namely strand-biased mutation and subsequent selection [reviewed in [11,17]]. These strand-biased mutation forces may be further classified into two basic categories: replication-driven mutation and transcription-driven mutation. Several mechanisms of replication-driven mutation have been proposed based on the asymmetrical structures of the replication forks [reviewed in [12]], including higher abundance of single-stranded gaps and nicks on the lagging strands that are prone to mismatch repair and cytosine deamination (leading to C-T transition) [10,18] and asymmetrical enzyme machineries that replicate the leading and lagging strands. Transcription-driven mutation has been proposed to include mutations associated with exposed non-transcribed strands during transcription and transcription-coupled repair [19]. The non-coding sequence (non-CDS) is under replication-related mutation pressure, and free from selection at the translation level. However, the transcribed non-CDS (upstream or downstream from the CDS) is still under transcription-driven mutation. The CDS, on the other hand, is affected by replication-driven mutation and transcription-driven mutation plus selection at the translation level.

These mutations undergo various kinds of selection, including the shaping of the signal sequences on the chromosomes [14] (see above). A universal and powerful selection is at the translation level, in which adverse mutations are eliminated or selected against. In addition, codon usage and amino acid usage preferences in combination also select optimal mutations at this level. The facts that codons usage in bacteria shows a preference for G over C (a translational selection) and that more genes (up to about 80% in some Gram-positive bacteria) are located on the leading strands than on the lagging strands of most bacterial chromosomes [20] automatically lead to G excess in the leading strands [21,22]. Moreover, selection pressure at the translation level may also produce biases in the usage of nucleotides, codons, and amino acids [23-27]. It has been noted that orthologs on the leading strands show lower rates of divergence than those on the lagging strands among various bacteria; this is a reflection of lower mutation pressure on the leading strand [28]. In many cases, these strand biases are considerable, and may be used to predict the replicating strand location of particular CDS with surprising accuracy [27].

The effect of mutations and subsequent selections on base composition skews cannot be readily separated in analysis. In general, the combined effect of replication-driven mutation plus subsequent selection is treated collectively as 'replication-associated pressure' (RAP), and the combined effect of transcription-driven mutation plus subsequent selection at the translation level as 'transcription-associate pressure' (TAP). While RAP is directly reflected in the overall skew, the effect of TAP depends on relative distribution of the CDS on the two replicating strands. If CDS are equally distributed between the leading and lagging strands, the effect of TAP on base composition skews is nil, and if CDS are present exclusively on one replicating strand, the TAP effect is total.

The TAP effect exerted on CDS on either replicating strand is equal, whereas the RAP effect has an opposite directionality on CDS on two replicating strands. Thus, the base composition skews of CDS on the two replicating strands may be used to extract the effects of RAP and TAP. This general principle has been applied by Lobry and Sueoka [29] to detect and assess RAP and TAP in 43 bacterial chromosomes using a graphic approach. These graphically deduced RAP and TAP were for GC and AT skews combined together. It was concluded that these two forces were most evident in the weakly selected third codon position and in intergenic regions. The authors noted that the directions of the two effects are almost universal (with some exceptions), resulting in G and T excess in the leading strands, which was compatible with the hypothesis of excess of cytosine deamination in the single-stranded state during DNA replication [11]. In fact the authors modeled their analysis based on C-T transitions and attributed any non-conformity to the effect of TAP.

In this study, using the same general principle but with a more comprehensive mathematical approach, we evaluated the RAP and TAP for GC skews and AT skews in 185 bacterial chromosomes from 11 phyla. The results show diverse and distinct RAP and TAP patterns among different families of the bacterial chromosomes, and each GC and AT skew-shaping force may be very different. While all the chromosomes are under significant TAP, a portion of them is under no or little RAP. Some bacteria (*e.g*., Firmicutes and proteobacteria) exhibit high RAP and high TAP, some (*e.g*., Chlamydiae) exhibit only significant RAP and little or no TAP, and a few (*e.g*., Cyanobacteria) exhibit none of either. Analysis of the RAP and TAP shows that the cytosine deamination may be important for RAP in some bacteria such as proteobacteria, but not in TAP. Instead, there appears to be significant involvement of transversion in the generation of base composition skews.

Our study shows that chromosomes that exhibit high base composition skews generally possess high TAP and RAP. Moreover, the trends and magnitudes of the skews can be correlated to the size and G+C contents of the chromosomes. This is in line with the notion that the base composition skews and their underlying mechanisms are important to the shaping of the bacterial chromosomes during evolution.

## Results

### *χG vs. χA*: Clustering of related chromosomes

The overall base composition skews with respect to leading strands *vs*. lagging strands over the whole bacterial chromosome are designated *χG *(for GC skew) and *χA *(for AT skew). *χG *is defined as the total number of G minus the total number of C divided by the total number of G and C on the leading strands, and *χA *is defined as the total number of A minus the total number of T divided by the total number of A and T on the leading strands.

In order to assign the leading and lagging strands, the replication origin (*oriC*) and termination (*ter*) must be defined. *oriC *of only a few bacterial chromosomes has been experimentally determined. For the remaining majority, prediction of *oriC *has been based on several different parameters. For examples, Worning et al. [30] predicted the location of *oriC *using biased distribution of all oligonucleotides up to 8 bp, and Mackiewicz et al. [31] use three criteria – composition skew, location of *dnaA *gene, and distribution of DnaA box-like sequences – for *oriC *prediction. Here we have followed the basic method of Mackiewicz et al. [31] to predict *oriC*. Ninety-nine bacterial chromosomes with a predicted *oriC *were taken from Mackiewicz et al. [31]. From the available complete bacterial sequences, *oriC *was predicted for another 86 chromosomes. For circular chromosomes, the *ter *site was assigned to be directly opposite to *oriC*. For linear chromosomes, the ends are where replication terminates. In total, a total of 185 chromosomes representing 11 phyla [see Additional file 1] were included in this study. Of these bacteria, the largest Phyla are Firmicutes and Proteobacteria, and in many of the analyses, they were further subdivided into Classes. The sizes of the chromosomes ranged from 0.6 to 9.1 Mb (mean 3.4 Mb), and their G+C contents from 24 to 72% (mean 49 %).

*χG *and *χA *were calculated from the sequence of the 185 bacterial chromosomes. *χG *is statistically significant (*p *< 10^-3^, *χ*^2 ^test) for all except 12 bacteria, and *χA *are significant statistically (*p *< 10^-2^, *χ*^2 ^test) for all except 17 bacteria [see Additional file 1]. These exceptions include four of the five Cyanobacteria tested.

**Figure 1:**
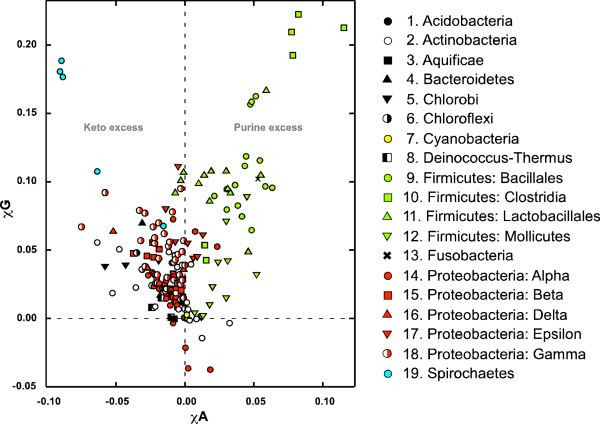
**Scatter-plot analysis of base composition skews – *χG *vs. *χA***. The *χG *values and *χA *values of 185 bacterial chromosomes are plotted against each other. The symbols for the 19 groups of bacterial chromosomes (1–19) are listed on the right. The 'Keto Excess' and the 'Purine Excess' trends in Quadrant I and II are indicated.

Figure 1 shows a scatter plot of *χG *vs. *χA *for the bacterial chromosomes. Interestingly, while the *χA *values spread from about -0.10 to 0.12, the *χG *values are mostly positive, ranging from -0.04 to about 0.24. The prevalence of positive *χG *values for most bacterial chromosomes is consistent with previous observations that G is more abundant in the leading strand of most bacterial chromosomes [12]. Only two Actinobacteria (Figure 1, white circles) and three ε-Proteobacteria (red circles) exhibited statistically significant negative *χG *values.

In the plot, related bacterial chromosomes tend to cluster together. For example, the Firmicute chromosomes (green symbols) are essentially all distributed in Quadrant I. Within the Firmicutes, members of the same Class also cluster together. Most other bacterial chromosomes are distributed in Quadrant II. Within Quadrant II, clustering is also seen for proteobacteria (red symbols) and its Classes. This is in accordance with the notion that the base distribution skews are evolutionally conserved.

### *χG vs. χA*: Two trends of distributions

The *χG *vs. *χA *plot also shows a general trend for the absolute values of these two values to increase in proportion (*r *= 0.72). From the denser central area, the chromosomes diverge in two general directions, one toward simultaneously increasing *χG *and *χA*, and the other toward increasing *χG *but decreasing *χA*. The former corresponds to 'purine excess' in the leading strand as noted by Freeman et al. [8] for nine bacterial chromosomes. Most of the chromosomes in this trend lie in Quadrant I, and belong to Firmicutes and also *F. nucleatum*. Of these, the clostridia chromosomes (green inverted triangles) have the highest *χA *and *χG *values. The other trend, in which *χG *varies in inverse proportion with *χA*, corresponds to 'keto excess' trend also noted by Freeman et al. [8]. Most of these chromosomes lie mainly in Quadrant II, but a few are in Quadrant IV. That related bacteria have the similar strengths of keto and purine excesses has also been noted by Song et al. [9] for 36 species examined.

### Base composition skews deviate more in non-CDS

For subsequent analysis, we separate the genome sequences into CDS (protein-coding sequences) and non-CDS (the remaining sequences). CDS constitutes the major portion of the bacterial chromosomes. In the 185 chromosomes investigated, the fractions of CDS range from 50.9% (*Sodalis glossinidius*) to 95.5% (*Candidatus Pelagibacter ubique*) with a mean of 86.2%.

CDS is susceptible to both RAP and TAP. Non-CDS is more complicated in that it contains both non-transcribed and transcribed regions (stable RNA genes and transcribed regions upstream and downstream of genes). The non-transcribed part is susceptible to RAP only, and the transcribed part is susceptible to both RAP and TAP (but without translation pressure). Unless transcription maps in non-CDS is available, it is impossible to investigate the components that shape the skews in non-CDS. In contrast, CDSs provide a simpler model for extraction of information regarding the operations of RAP and TAP in this study.

**Figure 2:**
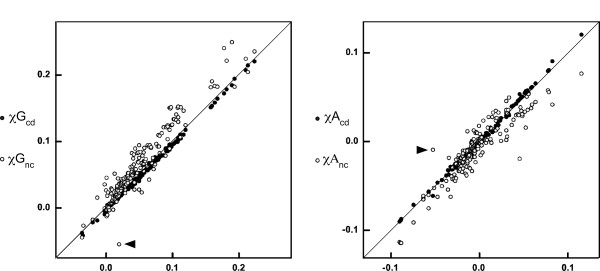
**Comparison of base composition skews in the CDS and non-CDS**. (A) The *χG*'s are plotted against *χG*_*cd*_'s (filled circles) and *χG*_*nc*_'s (open circles). (B) *χA*'s are plotted against *χA*_*cd*_'s (filled circles) and *χA*_*nc*_'s (open circles).

Thus, we break down *χG *and *χA *into those in the CDS (*χG*_*cd*_, *χA*_*cd*_), and those in the non-CDS (*χG*_*nc*_, *χA*_*nc*_). The scatter chart comparison (Figure 2, filled circles) shows that, for most chromosomes, *χG*_*cd *_and *χA*_*cd *_are nearly identical to *χG *and *χA*, respectively (mean differences of 2 × 10^-3 ^for both). This is not surprising, since CDS constitute the majority of bacterial genomes.

In contrast, *χG*_*nc *_and *χA*_*nc *_deviate noticeably more widely from *χG *and *χA*, respectively, for most bacterial chromosomes (Figure 2, open circles). Most (87%) of the *χG*_*nc *_values are higher than the corresponding *χG *values with a mean difference of 2 × 10^-2^. In contrast, *χA*_*nc *_is higher than the corresponding *χA *in only about 37% of the bacteria regardless of their phylogenetic groups. The deviations of skews in the non-CDS and the CDS presumably reflect the difference in the mutation pressures and selection pressures exerted on these sequences, which are expected to be lower in non-CDS.

### RAP and TAP are estimated from base composition skews in the CDS

The base composition skews in the CDS may be used to estimate RAP and TAP under the assumption that the effects of the two forces are independent of each other. This assumption is reasonable, because, considering the relatively low magnitude of the base composition skews, it is very unlikely that any nucleotide position is simultaneously affected by RAP and TAP.

The GC skew in the CDS on the leading strand (designated *σG*_*d*_) and on the lagging strand (designated *σG*_*g*_) may be represented, respectively, as:

*σG*_*d *_= *σG*^*T *^+ *σG*^*R*^

*σG*_*g *_= *σG*^*T *^- *σG*^*R*^

where *σG*^*T *^and *σG*^*R *^are GC skews shaped by TAP and RAP in CDS, respectively.

From these, *σG*^*T *^and *σG*^*R *^may be derived as:

*σG*^*T *^= (*σG*_*d *_+ *σG*_*g*_)/2

*σG*^*R *^= (*σG*_*d *_- *σG*_*g*_)/2

Similarly, the AT skews generated by TAP (*i.e*., *σA*^*T*^) and RAP (*i.e*., *σA*^*R*^) may be derived from AT skews in the CDS on the leading (*i.e*., *σA*_*d*_) and lagging strand (*i.e*., *σA*_*g*_) as:

*σA*^*T *^= (*σA*_*d *_+ *σA*_*g*_)/2

*σA*^*R *^= (*σA*_*d *_- *σA*_*g*_)/2

It is noteworthy that *σG*_*d *_- *σG*_*g *_and *σA*_*d *_- *σA*_*g *_correspond to 'ΔGC skew' and 'ΔAT skew', respectively, described by Rocha and Danchin [32], which are defined as the difference between the average skews of the genes in the leading strand and those in the lagging strand.

### Patterns of base composition skew-shaping RAP and TAP among bacterial families

**Figure 3:**
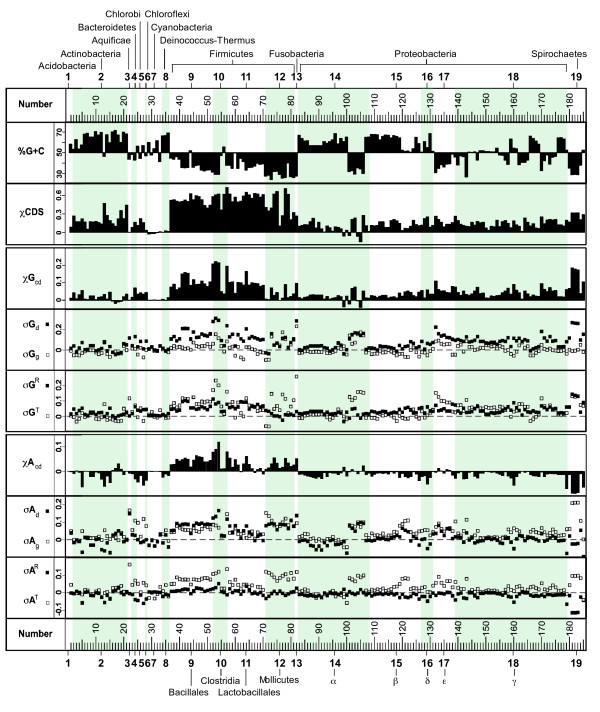
**The various genomic parameters relevant to the base composition skews of the bacterial chromosomes**. The bacteria are arranged in Genus (top) and Groups (1–19, Figure 1; shaded), and numbered [see Additional file 1 for complete list]. (Top panel) G+C contents and *χCDS*. (Middle panel) *χG*_*cd*_, *σG*_*d*_, *σG*_*g*_, *σG*^*R*^, and *σG*^*T*^. (Bottom panel) *χA*_*cd*_, *σA*_*d*_, *σA*_*g*_, *σA*^*R*^, and *σA*^*T*^. Filled squares are for the leading strands, and open squares are for lagging strands.

With the above equations, the base composition skews in CDS and the RAP and TAP effects for the 185 bacterial chromosomes were derived (Figure 3). *σG*_*d *_is statistically significant (*p *< 10^-2^, *χ*^2 ^test) in all bacterial chromosomes except for five [see Additional file 1], and *σG*_*g *_is significant in all except twelve. *σA*_*d *_is significant in all except thirteen, and *σA*_*g *_is significant in all except thirteen [see Additional file 1]. Statistically insignificant *σG*_*g *_and *σA*_*g *_values, however, should not necessarily be taken as an indication of a lack of a TAP on the base composition skews in CDS on the lagging strand (*CDS*_*g*_), but may reflect effect of the counteracting of RAP on the skews in these bacteria.

Different phyla exhibit distinct patterns of skews in base compositions and CDS (*χCDS*; see below), and within the same phylum different species tend to exhibit similar patterns. For example, the Firmicute chromosomes (Groups 9–12) have the highest *χCDS*, *χG*_*cd*_, and *χA*_*cd*_. In contrast, the Cyanobacterial chromosomes (Group 7) have essentially no *χCDS*, *χG*_*cd*_, or *χA*_*cd*_. Most phyla also display distinct patterns associated with the calculated effects of RAP and TAP on the base composition skews. Most strikingly, the Spirochaete chromosomes (Group 19) have large and approximately equal *σG*^*T *^and *σG*^*R *^values, together with large *σA*^*T *^and *σA*^*R *^values of opposite signs. In contrast, all these values are nearly zero in Actinobacterial chromosomes (Group 2).

To assess the general effects of RAP and TAP in seven larger phyla, their averaged *σG*^*T*^, *σG*^*R*^, *σA*^*T*^, and *σA*^*R *^values are calculated and listed in Table 1. From the list, some general trends may be seen. The *σG*^*T *^averages are very small (≤ 0.005) and vary widely in three phyla (Actinobacteria, Chlorobi, and Deinococcus-Thurmus), but are relatively large in the other four phyla, particularly in the Spirochaetes (0.096) and Firmicutes (0.075). The *σG*^*R *^averages are positive in all seven phyla and range from 0.001 (Cyanobacteria) to 0.096 (Spirochaetes). Therefore, it appears that there is a general trend of bias toward G excess for both RAP and TAP in most of the bacteria.

**Table 1:** Average base composition skews in *CDS *and RAP and TAP effects in seven phyla of bacteria*

	Actinobacteria	Chlorobi	Cyanobacteria	Deinococcus-Thermus	Firmicutes	Proteobacteria	Spirochaetes
*σG*_*d*_	**0.012**	**0.039**	**0.012**	**0.002**	**0.124**	**0.064**	**0.192**
	± 0.036	± 0.037	± 0.019	± 0.014	± 0.073	± 0.051	± 0.079
*σG*_*g*_	**-0.022**	**-0.034**	**0.009**	**-0.012**	**0.027**	**0.004**	**0.001**
	± 0.034	± 0.048	± 0.021	± 0.015	± 0.059	± 0.048	± 0.025

*σG*^*T*^	**(-0.005)**	**(0.002)**	**0.010**	**(-0.005)**	**0.075**	**0.034**	**0.096**
	± 0.030	± 0.043	± 0.020	± 0.012	± 0.059	± 0.046	± 0.035
*σG*^*R*^	**0.017**	**0.037**	**(0.001)**	**0.007**	**0.049**	**0.030**	**0.096**
	± 0.018	± 0.005	± 0.001	± 0.008	± 0.032	± 0.019	± 0.047

							

*σA*_*d*_	**-0.011**	**-0.010**	**-0.011**	**-0.011**	**0.070**	**0.005**	**-0.016**
	± 0.036	± 0.008	± 0.026	± 0.035	± 0.036	± 0.034	± 0.051
*σA*_*g*_	**0.012**	**0.077**	**-0.011**	**0.025**	**0.063**	**0.035**	**0.133**
	± 0.017	± 0.053	± 0.028	± 0.031	± 0.034	± 0.032	± 0.091

*σA*^*T*^	**(0.001)**	**0.033**	**-0.011**	**0.007**	**0.067**	**0.020**	**0.058**
	± 0.018	± 0.029	± 0.027	± 0.032	± 0.031	± 0.030	± 0.056
*σA*^*R*^	**-0.012**	**-0.043**	**(-0.000)**	**-0.018**	**(0.004)**	**-0.015**	**-0.075**
	± 0.022	± 0.024	± 0.001	± 0.006	± 0.016	± 0.013	± 0.047

*Standard deviations are listed below the averages. Absolute values of *σG*^*T*^, *σG*^*R*^, *σA*^*T*^, and *σA*^*R *^equal to or smaller than 0.005 are in parentheses.

The *σA*^*T *^averages are positive in all the phyla (0.001~0.067) except in Cyanobacteria (-0.011). In contrast, the *σA*^*R *^averages are negative (-0.012~-0.075) in five of the seven major phyla and near zero in the other two (Cyanobacteria and Firmicutes). Therefore, TAP is generally biased toward *A *excess in all the seven major phyla, while RAP is biased toward *T *excess in five major phyla and very low or non-existing in the other two.

### The effect of TAP depends on *χCDS*

*χG*_*cd *_may be quantitatively presented as:

χGcd=σGR+CDSdCDSd+CDSg⋅σGT−CDSgCDSd+CDSg⋅σGT,
 MathType@MTEF@5@5@+=feaafiart1ev1aaatCvAUfKttLearuWrP9MDH5MBPbIqV92AaeXatLxBI9gBaebbnrfifHhDYfgasaacH8akY=wiFfYdH8Gipec8Eeeu0xXdbba9frFj0=OqFfea0dXdd9vqai=hGuQ8kuc9pgc9s8qqaq=dirpe0xb9q8qiLsFr0=vr0=vr0dc8meaabaqaciaacaGaaeqabaqabeGadaaakeaaiiGacqWFhpWycqWGhbWrdaWgaaWcbaGaem4yamMaemizaqgabeaakiabg2da9iab=n8aZjabdEeahnaaCaaaleqabaGaemOuaifaaOGaey4kaSYaaSaaaeaacqWGdbWqcqWGebarcqWGtbWudaWgaaWcbaGaemizaqgabeaaaOqaaiabdoeadjabdseaejabdofatnaaBaaaleaacqWGKbazaeqaaOGaey4kaSIaem4qamKaemiraqKaem4uam1aaSbaaSqaaiabdEgaNbqabaaaaOGaeyyXICTae83WdmNaem4raC0aaWbaaSqabeaacqWGubavaaGccqGHsisldaWcaaqaaiabdoeadjabdseaejabdofatnaaBaaaleaacqWGNbWzaeqaaaGcbaGaem4qamKaemiraqKaem4uam1aaSbaaSqaaiabdsgaKbqabaGccqGHRaWkcqWGdbWqcqWGebarcqWGtbWudaWgaaWcbaGaem4zaCgabeaaaaGccqGHflY1cqWFdpWCcqWGhbWrdaahaaWcbeqaaiabdsfaubaakiabcYcaSaaa@664D@

where *CDS*_*d *_and *CDS*_*g *_are the total lengths of CDS on the leading and lagging strands, respectively, or

*χG*_*cd *_= *σG*^*R *^+ *χCDS*·*σG*^*T*^,

where χCDS=CDSd−CDSgCDSd+CDSg
 MathType@MTEF@5@5@+=feaafiart1ev1aaatCvAUfKttLearuWrP9MDH5MBPbIqV92AaeXatLxBI9gBaebbnrfifHhDYfgasaacH8akY=wiFfYdH8Gipec8Eeeu0xXdbba9frFj0=OqFfea0dXdd9vqai=hGuQ8kuc9pgc9s8qqaq=dirpe0xb9q8qiLsFr0=vr0=vr0dc8meaabaqaciaacaGaaeqabaqabeGadaaakeaaiiGacqWFhpWycqWGdbWqcqWGebarcqWGtbWucqGH9aqpdaWcaaqaaiabdoeadjabdseaejabdofatnaaBaaaleaacqWGKbazaeqaaOGaeyOeI0Iaem4qamKaemiraqKaem4uam1aaSbaaSqaaiabdEgaNbqabaaakeaacqWGdbWqcqWGebarcqWGtbWudaWgaaWcbaGaemizaqgabeaakiabgUcaRiabdoeadjabdseaejabdofatnaaBaaaleaacqWGNbWzaeqaaaaaaaa@47F8@

Similarly,

*χA*_*cd *_= *σA*^*R *^+ *χCDS*·*σA*^*T*^

Equations (5) and (6) are based on the assumptions that (*i*) the TAP effect (*σG*^*T *^and (*σA*^*T*^) is equal on the leading and lagging strands, and (*ii*) the RAP and TAP effects are independent. Such assumptions are supported by the results of Tillier and Collins [22] in their analysis of 12 bacterial species. Moreover, *σG*_*cd *_and *χA*_*cd *_values calculated from (5) and (6) differed from the actual values only slightly. The mean of errors is 3 × 10^-4 ^± 6 × 10^-4 ^(S. D.) for *χG*_*cd*_, and 2 × 10^-4 ^± 2 × 10^-4 ^(S. D.) for *χA*_*cd*_.

Equations (5) and (6) bring in the third main factor in determining the base composition skews in the CDS of bacterial chromosomes, *χCDS*. The *χCDS *values vary between -0.15 to 0.74 with a mean of 0.23 among the 185 bacterial chromosomes. When *χCDS *approaches zero (equal distribution of CDS on the replicating strands), the skews are contributed to by RAP only.

The Firmicutes have the highest *χCDS *(0.11~0.74), which, in combination with moderately high *σG*^*R *^and *σA*^*R *^values, produce the highest *χG*_*cd *_and *χA*_*cd *_among all the bacterial groups (Figure 3). The high *χCDS *values in the Firmicutes have been noted to be associated with the presence of a *polC *gene in the genome [33]: Chromosome containing both *polC *and *dnaE *have an average *χCDS *of 0.78, whereas for chromosomes containing only *dnaE *have an average *χCDS *of 0.58. The reason for this correlation is not clear.

### Both TAP and RAP correlate positively with *χG*_*cd *_and *χA*_*cd*_

**Figure 4:**
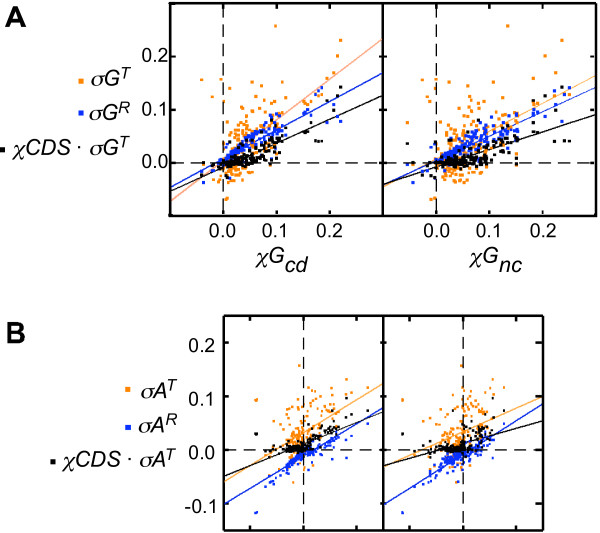
**Correlation analysis of RAP and TAP**. (A) Correlation between *σG*^*R *^(blue symbols), *σG*^*T *^(red symbols), and *χCDS*·*σG*^*T *^(black symbols) to *χG*_*cd *_(left panel) and to *χG*_*nc *_(right panel). (B) Correlation between *σA*^*R *^(blue symbols), *σA*^*T *^(red symbols), and *χCDS*·*σA*^*T *^(black symbols) to *χA*_*cd *_(left panel) and to *χA*_*nc *_(right panel). The slopes (*m*) and correlation coefficients (*r*) of the regression lines are listed in Table 2.

The relative contributions of RAP and TAP to the base composition skews in the CDS were compared by plotting *χG*_*cd *_and *χA*_*cd *_against *σG*^*R*^, *σG*^*T*^, and *χCDS*·*σG*^*T*^, and against *σA*^*R*^, *σA*^*T*^, and *χCDS*·*σA*^*T*^, respectively (Figure 4; Table 2). *χG*_*cd *_is strongly correlated to *σG*^*R *^(*r *= 0.88), and the linear regression line intersects the axes near the origin with a slope of 0.54. *χG*_*cd *_is only moderately correlated with *σG*^*T *^(*r *= 0.64), but is strongly correlated with *χCDS*·*σG*^*T *^(*r *= 0.84) as expected. The *χG*_*cd *_vs. *χCDS*·*σG*^*T *^regression line also intersects near the origin, and the slope of this line is 0.45.

**Table 2:** Contribution of RAP and TAP to the base composition skews in CDS (derived from Figure 4)

	*χG*_*cd*_		*χG*_*nc*_	
	
	*m*	*r*	*m*	*r*
*σG*^*R*^	0.55	0.85	0.45	0.84
*σG*^*T*^	0.59	0.51	0.42	0.44
*χCDS*·*σG*^*T*^	0.44	0.79	0.30	0.66

	*χA*_*cd*_		*χA*_*nc*_	
	
	*m*	*r*	*m*	*r*

*σA*^*R*^	0.55	0.86	0.58	0.86
*σA*^*T*^	0.70	0.59	0.55	0.45
*χCDS*·*σA*^*T*^	0.45	0.81	0.35	0.60

*m*, slope of linear regression line; *r*, correlation coefficient

Similarly, *χA*_*cd *_is also strongly correlated to *σA*^*R *^(*r *= 0.85). The linear regression line for *χA*_*cd *_*vs*. *σA*^*R *^passes near the origin, and has also a slope of 0.60. *χA*_*cd *_is weakly correlated with *σA*^*T *^(*r *= 0.49), but is strongly correlated with *χCDS*·*σA*^*T *^(*r *= 0.72) as expected. The *χA*_*cd *_vs. *χCDS*·*σA*^*T *^linear regression line also intersect near the origin with a slope of 0.40.

These results indicate that the relative contribution to either *χA*_*cd *_or *χG*_*cd *_by RAP and TAP (after *χCDS *attenuation) is approximately 60% and 40%, respectively, across the bacterial spectrum. These average values, however, are not good reflections of RAP and TAP in individual species, which may deviate from these average ratios significantly.

### The skews in the non-CDS is more correlated to RAP than to TAP

Regression analysis of the effect of RAP and TAP on the base composition skews in the non-CDS (Figure 4) shows that *χG*_*nc *_and *χA*_*nc *_are not only strongly correlated with *σG*^*R *^(*r *= 0.88) and *σA*^*R *^(*r *= 0.86), respectively, but also moderately correlated to *σG*^*T *^(*r *= 0.51) and *σA*^*T *^(*r *= 0.34), respectively, and moderately correlated to *χCDS*·*σG*^*T *^(*r *= 0.72) and *χCDS*·*σA*^*T *^(*r *= 0.49), respectively. The latter two sets of correlation presumably are due to the presence of transcribed sequences in the set of non-CDS, which would be under transcription-driven pressure, but not translation-driven pressure. The transcribed non-CDS cannot be readily separated from the non-transcribe non-CDS, and therefore the RAP and TAP on the two groups of sequences cannot be separately evaluated from Figure 4.

### The RAP and TAP trend analysis disfavors the cytosine deamination model

The RAP and TAP analysis may be used to examine the cytosine deamination model [27,34,35], which proposes that base composition skew is generated by preferred deamination of cytosine in single-stranded DNA such as the lagging strands during replication and the sense strands during transcription [18,36]. If strand-biased cytosine deamination plays a major role in shaping base composition skews during replication and transcription, the result of C to T transition would be reflected as a negative correlation between *σG*^*R *^and *σA*^*R *^and between *σG*^*T *^and *σA*^*T*^, respectively.

**Figure 5:**
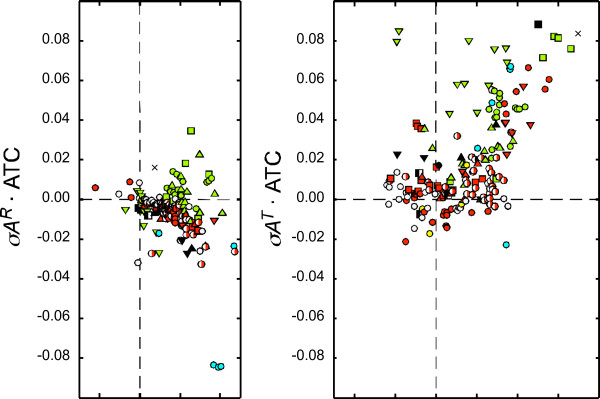
**Examination of cytosine deamination effect**. G+C content-adjusted *σG*^*R *^(*σG*^*R*^·GCC) is plotted against A+T content-adjusted *σA*^*R *^(*σA*^*R*^·ATC; left panel), and G+C content-adjusted *σG*^*T *^(*σG*^*T*^·GCC) is plotted against A+T content-adjusted *σA*^*T *^(*σA*^*T*^·ATC; right panel) for the 185 bacterial chromosomes. The symbols are as in Figure 1.

Correlation analysis between these forces with a weight adjustment for different G+C contents in the 185 chromosomes is shown in Figure 5. The analysis shows a weak *negative *correlation between weight-adjusted *σG*^*R *^and *σA*^*R *^(*m *= -0.29, *r *= 0.34, *p *< 0.01; left panel). For individual clades, negative correlation is strong in the α-, β-, and γ-Proteobacteria (*m *= -0.46~-0.64, *r *= -0.68~-0.87, *p *< 0.01), moderate in the ε-Proteobacteria (*m *= -0.65, *r *= -0.74; *p *= 0.06), and insignificant in the δ-Proteobacteria (*p *= 0.15). Of all the Firmicutes, only the Mollicutes shows a (negative) significant correlation (*m *= -1.43, *r *= -0.68, *p *= 0.02). No significant correlation exists in other phyla, which, in some cases (*e.g*., δ-Proteobacteria) is due to the small sample size. Therefore, the cytosine deamination model appears to be only applicable to RAP in the Proteobacteria (except δ-Proteobacteria) and the Mollicutes.

A weak *positive *correlation was seen between weight-adjusted *σG*^*T *^and *σA*^*T *^in the 185 chromosomes (*m *= 0.42, *r *= 0.54, *p *< 0.01; right panel). Examination of individual phyla shows only significant positive correlations (*m *= 0.97~2.01, *r *= 0.75~0.94, *p *ranging from < 0.01 to 0.05) in α-Proteobacteria and ε-Proteobacteria, and Clostridia in Firmicutes. No significant correlation exists in other phyla, which, in some cases, is due to the small sample size. The lack of negative correlation here indicates that the cytosine deamination model does not play a major role in shaping TAP in most if not all bacteria.

### RAP at three codon positions reveals lacks of RAP in some bacteria

The three positions of codons are under different selection pressures at the translation level. The third position is 4-way or 2-way degenerate for most amino acids, and enjoys the largest freedom for synonymous substitutions. The G+C content of a bacterial chromosome is principally shaped by the G+C content at this position [37,38]. The G+C content of the other two positions correlate only weakly with the G+C content of the chromosome, but still exhibit biased preference for different bases: a significant overrepresentation of G and underrepresentation of T at the first position and overrepresentation of A and T and underrepresentation of G at the second position. In our tabulation of the 185 bacterial chromosomes, frequencies of occurrence are 0.35 and 0.17 of G and T, respectively, at the first position, 0.30, 0.30, and 0.17 of A, T, and G, respectively, at the second position. These biases constitute part of the selection at the level of translation.

**Figure 6:**
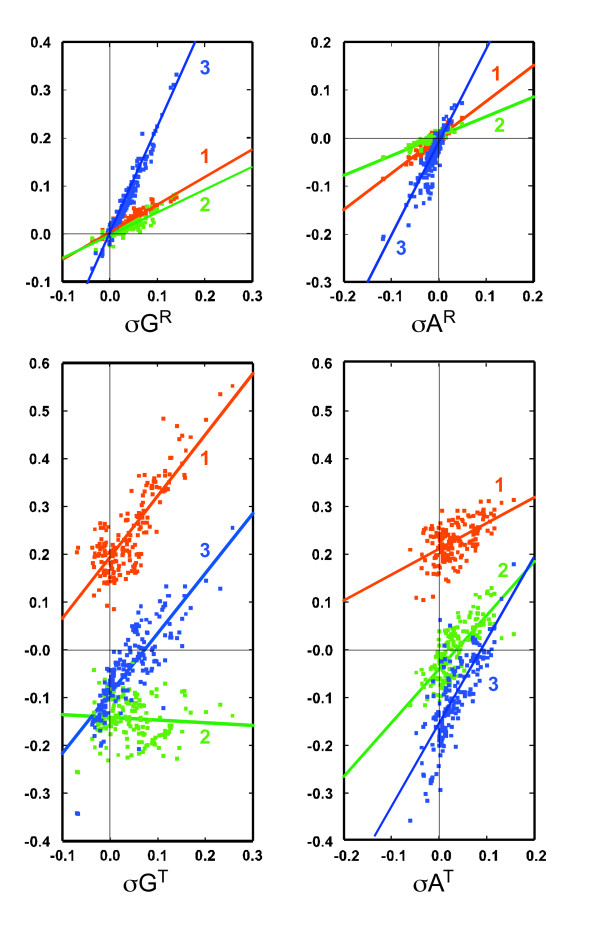
**RAP and TAP at the three positions of codons**. *σG*^*R*^, *σA*^*R*^, *σG*^*T*^, and *σA*^*T *^at three positions of codons (*y*-axis) are plotted against the overall *σG*^*R*^, *σA*^*R*^, *σG*^*T*^, and *σA*^*T*^, respectively (*x*-axis). Red, position 1; green, position 2; blue, position 3. The linear regression trend lines for each position are depicted.

We investigated RAP and TAP at the three codon positions in all the bacterial chromosomes. The plots of *σG*^*R *^and *σA*^*R *^at these positions against the overall *σG*^*R *^and *σA*^*R *^(Figure 6, top two panels) showed strong linear correlation between them (*r *= 0.86~0.98). The *σG*^*R *^and *σA*^*R *^effects (reflecting RAP involved in GC and AT skews) are the strongest at the third codon position as expected. The effect is significantly lower at the other two positions. Notably the three trend lines intersect at the origin, indicating that some bacteria possess nearly no overall *σG*^*R *^and *σA*^*R *^as well as *σG*^*R *^and *σA*^*R *^at all three positions. This means that, for these bacteria, there is very little or no RAP.

### TAP at three codon positions reveals omnipresence of TAP in all bacterial chromosomes

In contrast, in the plots of *σG*^*T *^and *σA*^*T *^at the three positions against the overall *σG*^*T *^and *σA*^*T*^, none of the three linear trend lines intersect at the origin (Figure 6, bottom two panels). Even for the bacterial chromosomes that exhibit no or little overall *σG*^*T *^and *σA*^*T*^, their *σG*^*T *^and *σA*^*T *^values at the three codon positions are far from zero. In these chromosomes, TAP is all positive on the first codon position, but is cancelled out by the negative effect on the other two positions. The lack of any all-zero case indicates the presence of considerable TAP in all the bacterial chromosomes.

The *σG*^*T *^correlation plot (bottom left panel) showed the strongest TAP effect at the first and third positions (*r *= 0.82, 0.82). The strong correlation at the more relaxed third position is expected. The strong and all positive effect at the first codon may be attributed to the selection for overrepresentation (by 60%) of G at that position in all the bacteria. There is essentially no correlation (*r *= -0.08) at the second position. *σG*^*T *^at the second position is negative regardless of the overall *σG*^*T*^, presumably reflecting the underrepresentation (by 32%) of G at this position.

In the *σA*^*T *^correlation plot (bottom right panel), all three positions exhibit a moderately strong linear correlation (*r *= 0.55~0.79). The all positive *σA*^*T *^values at the first position presumably reflect the underrepresentation of T (by 32%) at this position (see above).

### G+C content is strongly correlated with base composition skew

Examining the skews and other parameters of the 185 chromosomes (Figure 3) revealed a number of exceptional cases that exhibit skew patterns atypical for the particular clade. Interestingly, these atypical skews are accompanied by atypical G+C contents. For example, the six species of *Rickettsiales *(three *Rickettsia *species, two *Wolbachia *species, and *Candidatus Pelagibacter ubique*; chromosome 101~106) display atypical skews parameters among the α-proteobacteria. They also stand out in this clade as displaying unusually low G+C contents (29.0–35.2% *vs*. an average of 61.5%). Moreover, two closely related spirochaetes, *Treponema denticola *and *Treponema pallidum *(chromosome number 184 and 185) differ greatly in skew parameters as well as G+C contents.

**Figure 7:**
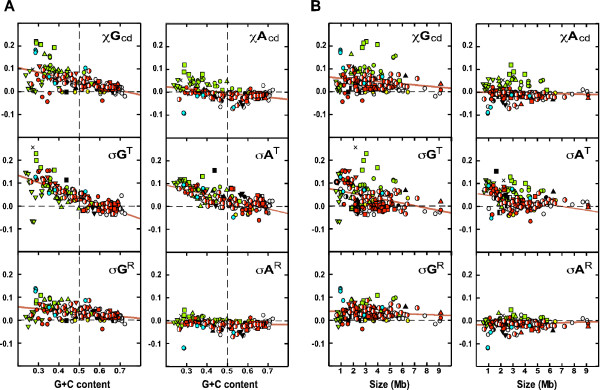
**Correlation between the G+C content and the size of the chromosomes and the effects of RAP and TAP**. σGcdR
 MathType@MTEF@5@5@+=feaafiart1ev1aaatCvAUfKttLearuWrP9MDH5MBPbIqV92AaeXatLxBI9gBaebbnrfifHhDYfgasaacH8akY=wiFfYdH8Gipec8Eeeu0xXdbba9frFj0=OqFfea0dXdd9vqai=hGuQ8kuc9pgc9s8qqaq=dirpe0xb9q8qiLsFr0=vr0=vr0dc8meaabaqaciaacaGaaeqabaqabeGadaaakeaaiiGacqWFdpWCcqWGhbWrdaqhaaWcbaGaem4yamMaemizaqgabaGaemOuaifaaaaa@3387@, σGcdT
 MathType@MTEF@5@5@+=feaafiart1ev1aaatCvAUfKttLearuWrP9MDH5MBPbIqV92AaeXatLxBI9gBaebbnrfifHhDYfgasaacH8akY=wiFfYdH8Gipec8Eeeu0xXdbba9frFj0=OqFfea0dXdd9vqai=hGuQ8kuc9pgc9s8qqaq=dirpe0xb9q8qiLsFr0=vr0=vr0dc8meaabaqaciaacaGaaeqabaqabeGadaaakeaaiiGacqWFdpWCcqWGhbWrdaqhaaWcbaGaem4yamMaemizaqgabaGaemivaqfaaaaa@338B@, *χG*_*cd*_, σAcdT
 MathType@MTEF@5@5@+=feaafiart1ev1aaatCvAUfKttLearuWrP9MDH5MBPbIqV92AaeXatLxBI9gBaebbnrfifHhDYfgasaacH8akY=wiFfYdH8Gipec8Eeeu0xXdbba9frFj0=OqFfea0dXdd9vqai=hGuQ8kuc9pgc9s8qqaq=dirpe0xb9q8qiLsFr0=vr0=vr0dc8meaabaqaciaacaGaaeqabaqabeGadaaakeaaiiGacqWFdpWCcqWGbbqqdaqhaaWcbaGaem4yamMaemizaqgabaGaemivaqfaaaaa@337F@, σAcdR
 MathType@MTEF@5@5@+=feaafiart1ev1aaatCvAUfKttLearuWrP9MDH5MBPbIqV92AaeXatLxBI9gBaebbnrfifHhDYfgasaacH8akY=wiFfYdH8Gipec8Eeeu0xXdbba9frFj0=OqFfea0dXdd9vqai=hGuQ8kuc9pgc9s8qqaq=dirpe0xb9q8qiLsFr0=vr0=vr0dc8meaabaqaciaacaGaaeqabaqabeGadaaakeaaiiGacqWFdpWCcqWGbbqqdaqhaaWcbaGaem4yamMaemizaqgabaGaemOuaifaaaaa@337B@, and *χA*_*cd *_are plotted against the G+C content (A), and the size (B) of the chromosomes. The linear regression lines are in red. The symbols for the bacterial chromosomes are as in Figure 1.

To investigate the correlation between the G+C contents and the skew parameters, the G+C content of the bacteria was plotted against the skew parameters (Figure 7A). The results show that G+C content is strongly and inversely correlated with *σG*^*T *^(*r *= -0.77), but loosely and inversely correlated with *σG*^*R *^(*r *= -0.40). *χG*_*cd *_also shows an inverse correlation with G+C content (*r *= -0.58). These inverse correlations are most evident in the Actinobacteria (white-filled circles) and Proteobacteria (red symbols), but are looser in Firmicutes (green symbols).

G+C content is strongly and inversely correlated with *σA*^*T *^(*r *= -0.71) but not with *σA*^*R *^(*r *= -0.09; *p *> 0.05). *χA*_*cd *_is only weakly and inversely correlated with G+C content (*r *= 0.40). G+C content is also loosely correlated with the size of the bacterial chromosomes (*r *= 0.55; data not shown) as previously noted [39]. Therefore, correlation between the chromosomal size and the skew parameters were also examined (Figure 7B). The analysis shows an insignificant correlation between the chromosomal size and *σG*^*R *^(*r *= -0.12) and *σA*^*R *^(*r *= -0.12), but a weak negative correlation between the chromosomal size and *σG*^*T *^(*r *= -0.33) and *σA*^*T *^(*r *= -0.35).

There appears to be a general trend toward decreasing magnitudes of the skew parameters with increasing chromosome size. The GC skew parameters converge from positive values toward zero; whereas the AT skew parameters converge from positive and negative values toward zero. This seems to suggest that larger bacterial chromosomes are under lower RAP and TAP.

## Discussion

### Omnipresence of TAP

The present study of the base composition skews reveals widely variable patterns of skews as well as the underlying shaping forces, suggesting extensive diversification of the mutation and selection spectra during evolution of the bacterial chromosomes. Analyzing base substitutions between orthologs in 33 closely related strains in 6 clades, Rocha et al. [40] have previously reached similar conclusions. Based on this, these authors proposed that the skew shaping process is multifactorial. The same conclusion may be drawn from the current study.

The RAP and TAP analysis at the three codon positions (Figure 6) reveals interesting contrasts between the two. Most remarkable is the omnipresence of TAP, in contrast to the absence of RAP in portions of the bacteria. The analysis also shows that TAP is (at least partly) contributed by selection pressure at the translational level that generates the biased base composition at the first two codon positions. It implies that the apparent lack of TAP in some bacterial chromosomes does not reflect the absence of it, but rather cancellation among the effect on the three positions.

### Comparison with previous studies

The basic principle of deducing RAP and TAP by comparison of base composition skews in the CDS on the two replicating strands used in this study is similar to that employed by Mackiewicz et al. [26] and Lobry and Sueoka [29].

Mackiewicz et al. [26] performed 'detrended DNA walks' on seven bacterial chromosomes, which displayed base composition skews on a two-dimensional plot. DNA walks on nucleotides in the CDS on two complementary strands of the chromosomes were added or subtracted. Addition of the skews would cancel the effect of RAP, thus revealing other mutation and selection effect (essentially equivalent to RAP). Subtraction, on the other hand, would cancel the effect of TAP, and leaving RAP. The subtraction (RAP) curve would exhibit a sign switch whereas the addition (TAP) curve would exhibit a maximum and minimum at the origin and terminus of replication. The results of such analyses were available for the chromosomes of *B. subtilis *[26] and *B. burgdorferi *[41].

**Figure 8:**
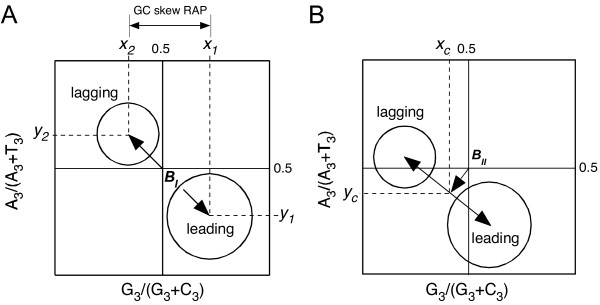
**Graphic analysis of TAP and RAP [29]**. A schematic representation of AT skews at the third position of codon, A_3_/(A_3_+T_3_), plotted against GC skews at the third position of codon, G_3_/(G_3_+C_3_), for all CDS in a genome. The circles represent those CDS on the lagging strands (usually smaller numbers) and the leading strand (usually larger numbers). Line *B*_*II *_connecting the average points, (*x*_2_, *y*_2_) and (*x*_1_, *y*_1_), of these two populations represents the average distance between the populations. (A) Scenario I – presence of RAP only (no TAP). No TAP is present. RAP creates G and T excess in the leading strand (and A and T excess in the lagging strands), thus separating the two populations in the indicated direction. The relative strength of RAP corresponds to the length of line B1=(x2−x1)2+(y2−y1)2
 MathType@MTEF@5@5@+=feaafiart1ev1aaatCvAUfKttLearuWrP9MDH5MBPbIqV92AaeXatLxBI9gBaebbnrfifHhDYfgasaacH8akY=wiFfYdH8Gipec8Eeeu0xXdbba9frFj0=OqFfea0dXdd9vqai=hGuQ8kuc9pgc9s8qqaq=dirpe0xb9q8qiLsFr0=vr0=vr0dc8meaabaqaciaacaGaaeqabaqabeGadaaakeaacqWGcbGqdaWgaaWcbaGaeGymaedabeaakiabg2da9maakaaabaGaeiikaGIaemiEaG3aaSbaaSqaaiabikdaYaqabaGccqGHsislcqWG4baEdaWgaaWcbaGaeGymaedabeaakiabcMcaPmaaCaaaleqabaGaeGOmaidaaOGaey4kaSIaeiikaGIaemyEaK3aaSbaaSqaaiabikdaYaqabaGccqGHsislcqWG5bqEdaWgaaWcbaGaeGymaedabeaakiabcMcaPmaaCaaaleqabaGaeGOmaidaaaqabaaaaa@42E1@. Line *B*_*I *_(double arrows) intercepts the midpoint (0.5, 0.5). (B) Scenario II – presence of both RAP and TAP. TAP exerts asymmetric effect on CDSs on both the leading and lagging strands, thus pushing *B*_*I *_away from the midpoint (0.5, 0.5). The relative strength of TAP corresponds to *B*_*II *_(arrow), the distance between *B*_*I *_and the midpoint. Modified from Lobry and Sueoka [29].

In their analysis of 43 bacterial chromosomes, Lobry and Sueoka [29] plotted GC and AT skews at the third codon positions on the two replicating strands against each other (Figure 8), from which RAP and TAP were derived graphically. The presence of RAP separates these skews on the leading and lagging strands into two distinct groups, and the distance between the centers (averages) of the two groups (Figure 7A, line *B*_*I*_) is taken to represent RAP. The authors assumed that, in the absence of TAP, *B*_*I *_would intercept the midpoint (0.5, 0.5) (Figure 8A), and that a deviation from that (Figure 8B, line *B*_*II*_) would represent TAP.

It is noteworthy that the relative RAP and TAP thus deduced were for both GC and AT skews combined. They may further be broken down into RAP and TAP specific for GC and AT skews by applying vector analysis, *i.e*., RAP for GC and AT skews corresponding to *y*_1 _- *y*_2 _and *x*_1 _- *x*_2_, respectively (Figure 8A); and 'TAP' for GC and AT skews corresponding to *y*_*c *_- 0.5 and *x*_*c *_- 0.5, respectively (Figure 8B). By applying this method on the original data of Lobry and Sueoka [29], we extracted relative RAP and TAP values from 32 bacterial chromosomes that are included in our study, and compare them to those derived mathematically in this study. Correlation is very high (*r *= 0.96 and 0.97, respectively) between their and our RAP values for both GC and AT skews. Correlation is also very high (*r *= 0.96) between their and our TAP values for AT skews, but slightly lower (*r *= 0.83) for GC skews.

### Asymmetric cytosine deamination model

Our RAP *vs*. TAP analysis (Figure 5) shows that the cytosine deamination model may be applicable to RAP in a number of clades, but not a major contributor to TAP in all bacteria. This suggests that, if the model is applicable, the exposed single strands during replication and during transcription may differ in length, binding proteins (*e.g*., single-strand binding proteins), and other characteristics.

Our conclusion appears to be different from previous studies that favor the cytosine deamination model for TAP [for example, [27,34,35]]. However, the previous studies are based on investigation of a small number of actively transcribed genes from Proteobacteria. For example, the conclusion of Francino and Ochman [34] was based a phylogenic comparison of an approximately 1.8-kb actively transcribed non-CDS in 12 *E. coli *strains. The *E. coli *chromosomes (No. 80~83) in the present study exhibit very low or statistically insignificant *χA *and *σA*^*T*^, but moderate *χG *and *σG*^*T*^, indicating a lack of a major contribution by cytosine deamination.

It is noteworthy that the number of bacterial genes highly expressed during exponential growth appears to be relatively small [42,43], and, there is evidence for the absence of cytosine deamination effect in the transcription of cryptic genes [35]. Thus, it is possible that either the small sample in the previous studies is not representative for chromosomes as a whole, or that the previously postulated cytosine deamination effect on TAP acts only on a small number of highly expressed genes, and represents only a minor fraction of overall TAP.

On the other hand, our analysis shows that cytosine deamination may play a significant role in RAP in α-, β-, γ-, and (to a lesser degree) ε-Proteobacteria, and Mollicutes. This is in general agreement with the base substitution analysis of Rocha et al. [40], which shows cytosine deamination is applicable in RAP in *Bordetella *(β-Proteobacteria), *E. coli *(γ-Proteobacteria), *Neisseria *(β-Proteobacteria), and *Streptococcus *(Firmicutes), but not in, *Bacillus *and *Staphylococcus *(two Firmicute clades), and *Rickettsia *(α-Proteobacteria). The latter, being intracellular parasites, may be considered an exceptional case.

In contrast to the situation in Proteobacteria, cytosine deamination cannot be applicable to RAP in most Firmicutes (except Mollicutes). This suggests a major difference in the state of single-stranded DNA exposed during replication in these two phyla of bacteria. In Proteobacteria, both strands of the chromosomes are replicated by DnaE. In contrast, the Firmicute genomes encode an additional replicase PolC [33], which is known to replicate the leading strand in *B. subtilis *[44]. Perhaps the two distinct systems generate single-stranded intermediates of very different states.

The distinctly different cytosine deamination effects between these two phyla of bacteria correspond to the separation of the base composition skews into the 'purine excess' trend in the Firmicutes and 'keto excess' trend in the Proteobacteria (Figure 1). The cytosine deamination model has been found to be the most likely cause of TAP in other studies [12,32]. However, the sample size (28) was considerably smaller than that in this study, and no clade-based analysis was performed.

### Skews and G+C content

**Figure 9:**
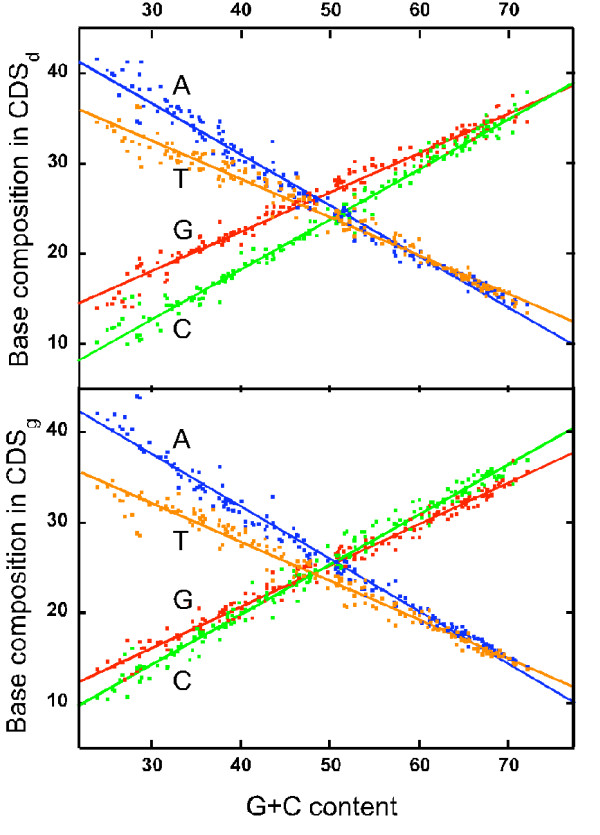
**Correlation between G+C content and base composition of CDSs**. The contents of four nucleotides in CDS_d _(upper panel) and CDS_g _(lower panel) in the 185 bacterial chromosomes are computed and plotted against their C+C content. The correlation coefficient for all the four trends are larger than 0.97. G content, red symbols; C content, red symbols; A content, blue symbols; T content, orange symbols.

In this study we found weak or no correlation between G+C content of the chromosomes and RAP on base composition skews, but a relatively strong negative correlation between G+C content and TAP on both GC and AT skews (Figure 6A). This is in line with the facts that the chromosomes with low G+C contents are mainly those of Firmicutes, and that the TAPs for both GC and AT skews (*σG*^*T *^and *σA*^*T*^) are highest in the Firmicute phylum (Figure 3; Table 1). The TAPs may also be analyzed by examining the relationship between G+C content and base composition in CDSs in the bacterial chromosomes (Figure 9), which shows that the *G *and *C *contents or *A *and *T *contents in CDSs do not vary in the same proportion to the G+C content of the chromosomes. At lower G+C contents, there is a distinct bias toward more *G*s than *C*s and more *A*s than *T*s (*i.e*., more purines than pyrimidine) in CDS on both replicating strands. At higher G+C contents, the trends are reversed albeit with lower deviations from the norms. These four biased trend lines correspond to the TAPs, and are highly correlated with G+C content (*r *> 0.97). This is in accordance with the linear correlation between *σG*^*T *^or *σA*^*T *^and G+C content (Figure 6B).

### Chromosomal sizes and skews

Because of the positive (albeit loose) correlation between the G+C content and size of bacterial chromosomes [39], it is not surprising to find that there is also a weak negative correlation between the chromosomal size and the TAPs (*σG*^*T *^and *σA*^*T*^; Figure 6B). However, there is an under-representation of larger bacterial chromosomes in the current set of sequenced genomes. It is possible that the convergence towards zero skews, TAPs, and RAPs observed in the larger chromosomes (Figure 6B) may be due to the small sample of large chromosomes. This remains to be investigated when more large bacterial chromosomes are sequenced.

On the other hand, the diminishing skews, TAPs, and RAPs in large chromosomes may be real and reflect an evolutional trend. It is reasonable to assume that the larger chromosomes have generally evolved from smaller ones (except for reductive evolution in parasites) by acquiring extra genes necessary for more complex structures (through differentiation) and contingency functions (*e.g*., secondary metabolism) that provide adaptability and a competition edge. Under this premise, the increase in gene number (and chromosomal size) is accompanied by an increase in G+C content and decrease in RAP, TAP, and base composition skews. If such an evolution trend is real, it suggests that RAP and TAP were stronger among ancestral bacteria.

The RAP and TAP analysis in this study may provide guidance for further bioinformatic and genetic investigations into the underlying principles for these mutation and selection forces. The best candidates for such investigations are probably closely relative species with distinct base composition parameters, such as the aforementioned *Rickettsiales *chromosomes (number 101 – 106), which display remarkably low G+C contents and high *σG*^*R*^, *σG*^*T*^, and *σA*^*T *^compared to other α-proteobacterial chromosomes, and the chromosomes of two spirochaetes, *T. denticola *and *T. pallidum *(number 184 and 185) that differ greatly in G+C contents and *σA*^*T *^(opposite signs). Moreover, the chromosomes of *Moorella thermoacetica *and *Thermoanaerobacter tengcongensis *(number 55 and 56) are also unique among the Clostridia in displaying atypically low *χG*_*cd*_, *χA*_*cd*_, *σG*^*T*^, and *σA*^*T*^. All these provide opportunities for comparative investigation to uncover the underlying genetic elements.

## Conclusion

In summary we have analyzed the base composition skews and the underlying mutation/selection forces associated with replication and transcription among 185 bacterial chromosomes in 11 phyla. The diverse patterns that are characteristic for different clades provide clues to the evolution that shape these skews. The correlation among the skews, the G+C content, and the size of the chromosomes also hints at the direction of the trends of the evolution.

## Methods

### Genomic sequences and assignment of *oriC *and *ter *sites

The chromosomal sequences were taken from National Center for Biotechnology Information [54]. Prediction of the *oriC *location followed the basic procedure of Mackiewicz et al. [31] using two methods: (*i*) DNA *a*symmetry (*i.e*., sign switch site of either GC or AT skew) and (*ii*) location of *dnaA *gene. A putative *oriC *was assigned at the first base of *dnaA*, when the locations predicted by these two methods were within 7% of the length of the chromosome. Chromosomes with more than one *dnaA *homologs were excluded. The *ter *site was assigned to be directly opposite of *oriC *for circular chromosomes. For linear chromosomes (such as those of *Streptomyces *and *Borrelia*), the ends are the *ter *sites.

### Definitions and conventions

*A*, *T*, *G*, and *C *denote the numbers of these nucleotides in the sequence or replicon under consideration. Their locations on the leading and lagging strands are denoted by subscript *d *and *g*, respectively. Overall base composition skews with respect to the leading and lagging strands in a bacterial chromosome are designated with the symbol *χ*, and defined by χG=Gd−GgGd+Gg=Gd−CdGd+Cd
 MathType@MTEF@5@5@+=feaafiart1ev1aaatCvAUfKttLearuWrP9MDH5MBPbIqV92AaeXatLxBI9gBaebbnrfifHhDYfgasaacH8akY=wiFfYdH8Gipec8Eeeu0xXdbba9frFj0=OqFfea0dXdd9vqai=hGuQ8kuc9pgc9s8qqaq=dirpe0xb9q8qiLsFr0=vr0=vr0dc8meaabaqaciaacaGaaeqabaqabeGadaaakeaaiiGacqWFhpWycqWGhbWrcqGH9aqpdaWcaaqaaiabdEeahnaaBaaaleaacqWGKbazaeqaaOGaeyOeI0Iaem4raC0aaSbaaSqaaiabdEgaNbqabaaakeaacqWGhbWrdaWgaaWcbaGaemizaqgabeaakiabgUcaRiabdEeahnaaBaaaleaacqWGNbWzaeqaaaaakiabg2da9maalaaabaGaem4raC0aaSbaaSqaaiabdsgaKbqabaGccqGHsislcqWGdbWqdaWgaaWcbaGaemizaqgabeaaaOqaaiabdEeahnaaBaaaleaacqWGKbazaeqaaOGaey4kaSIaem4qam0aaSbaaSqaaiabdsgaKbqabaaaaaaa@4A2D@ and χA=Ad−AgAd+Ag=Ad−TdAd+Td
 MathType@MTEF@5@5@+=feaafiart1ev1aaatCvAUfKttLearuWrP9MDH5MBPbIqV92AaeXatLxBI9gBaebbnrfifHhDYfgasaacH8akY=wiFfYdH8Gipec8Eeeu0xXdbba9frFj0=OqFfea0dXdd9vqai=hGuQ8kuc9pgc9s8qqaq=dirpe0xb9q8qiLsFr0=vr0=vr0dc8meaabaqaciaacaGaaeqabaqabeGadaaakeaaiiGacqWFhpWycqWGbbqqcqGH9aqpdaWcaaqaaiabdgeabnaaBaaaleaacqWGKbazaeqaaOGaeyOeI0Iaemyqae0aaSbaaSqaaiabdEgaNbqabaaakeaacqWGbbqqdaWgaaWcbaGaemizaqgabeaakiabgUcaRiabdgeabnaaBaaaleaacqWGNbWzaeqaaaaakiabg2da9maalaaabaGaemyqae0aaSbaaSqaaiabdsgaKbqabaGccqGHsislcqWGubavdaWgaaWcbaGaemizaqgabeaaaOqaaiabdgeabnaaBaaaleaacqWGKbazaeqaaOGaey4kaSIaemivaq1aaSbaaSqaaiabdsgaKbqabaaaaaaa@4A1D@.

A chromosomal sequence is divided into two super sets, CDS and non-CDS. CDS represent all the protein coding sequences, and non-CDS the rest of the sequences (including stable RNA-coding sequences). Quantitative parameters specific for CDS and non-CDS bear a *cd *and *nc *subscript, respectively. *χG*_*cd *_and *χA*_*cd *_represent calculated base composition skew with respect to the leading and lagging strand in CDS only; and *χG*_*nc*_, and *χA*_*nc*_, in non-CDS only. For example, χGcd=Gdcd−GgcdGdcd+Ggcd=Gdcd−CdcdGdcd+Cdcd
 MathType@MTEF@5@5@+=feaafiart1ev1aaatCvAUfKttLearuWrP9MDH5MBPbIqV92AaeXatLxBI9gBaebbnrfifHhDYfgasaacH8akY=wiFfYdH8Gipec8Eeeu0xXdbba9frFj0=OqFfea0dXdd9vqai=hGuQ8kuc9pgc9s8qqaq=dirpe0xb9q8qiLsFr0=vr0=vr0dc8meaabaqaciaacaGaaeqabaqabeGadaaakeaaiiGacqWFhpWycqWGhbWrdaWgaaWcbaGaem4yamMaemizaqgabeaakiabg2da9maalaaabaGaem4raC0aa0baaSqaaiabdsgaKbqaaiabdogaJjabdsgaKbaakiabgkHiTiabdEeahnaaDaaaleaacqWGNbWzaeaacqWGJbWycqWGKbazaaaakeaacqWGhbWrdaqhaaWcbaGaemizaqgabaGaem4yamMaemizaqgaaOGaey4kaSIaem4raC0aa0baaSqaaiabdEgaNbqaaiabdogaJjabdsgaKbaaaaGccqGH9aqpdaWcaaqaaiabdEeahnaaDaaaleaacqWGKbazaeaacqWGJbWycqWGKbazaaGccqGHsislcqWGdbWqdaqhaaWcbaGaemizaqgabaGaem4yamMaemizaqgaaaGcbaGaem4raC0aa0baaSqaaiabdsgaKbqaaiabdogaJjabdsgaKbaakiabgUcaRiabdoeadnaaDaaaleaacqWGKbazaeaacqWGJbWycqWGKbazaaaaaaaa@620B@, where the superscript *cd *denotes the bases in CDS.

The base composition skew in CDS is denoted by the symbol *σ*. Combined with the replicating strand designations, *σG*_*d *_and *σG*_*g *_denote GC skews in the CDS on the leading and lagging strands, respectively. *σA*_*d *_and *σA*_*g *_are similarly defined.

*χCDS*, a measure of the skew of distribution of CDSs with respect to the replicating strands, is defined as: χCDS=CDSd−CDSgCDSd+CDSg=CDSd−CDSgCDS
 MathType@MTEF@5@5@+=feaafiart1ev1aaatCvAUfKttLearuWrP9MDH5MBPbIqV92AaeXatLxBI9gBaebbnrfifHhDYfgasaacH8akY=wiFfYdH8Gipec8Eeeu0xXdbba9frFj0=OqFfea0dXdd9vqai=hGuQ8kuc9pgc9s8qqaq=dirpe0xb9q8qiLsFr0=vr0=vr0dc8meaabaqaciaacaGaaeqabaqabeGadaaakeaaiiGacqWFhpWycqWGdbWqcqWGebarcqWGtbWucqGH9aqpdaWcaaqaaiabdoeadjabdseaejabdofatnaaBaaaleaacqWGKbazaeqaaOGaeyOeI0Iaem4qamKaemiraqKaem4uam1aaSbaaSqaaiabdEgaNbqabaaakeaacqWGdbWqcqWGebarcqWGtbWudaWgaaWcbaGaemizaqgabeaakiabgUcaRiabdoeadjabdseaejabdofatnaaBaaaleaacqWGNbWzaeqaaaaakiabg2da9maalaaabaGaem4qamKaemiraqKaem4uam1aaSbaaSqaaiabdsgaKbqabaGccqGHsislcqWGdbWqcqWGebarcqWGtbWudaWgaaWcbaGaem4zaCgabeaaaOqaaiabdoeadjabdseaejabdofatbaaaaa@5706@.

The statistical significance of the calculated skews was estimated by binomial distribution probability and a *χ*^2 ^test.

### Data charting and statistic analysis

The processed data were charted and statistically analyzed using Aabel (version 2.1, Gigawiz) running under Mac OS X (version 10.4.8) on a PowerMac (Apple).

The complete analytical data of the 185 chromosomes are available in Additional file 1.

## Abbreviations

CDS, coding sequence; non-CDS, non-coding sequence; *CDS*_*d*_, coding sequence on the leading strand; *CDS*_*g*_, coding sequence on the lagging strand; RAP, replication-associated pressure; TAP, transcription-associated pressure

## Authors' contributions

CC carried out all the computation and participated in data analysis. CWC conceived of this study and participated in data analysis. Both authors contributed in the writing and revision of the manuscript, and approved its final form.

## Supplementary Material

Additional file 1 Complete list and analytical data of the 185 bacterial chromosomes used in this study. The table lists the bacterial chromosomes analyzed in this study and shows all the data obtained from computations, which are used in deriving the arguments and conclusions in this paper. Click here for file
