# Supplementary material for: Quantitative analysis of mutation and selection pressures on base composition skews in bacterial chromosomes
Source: BMC Genomics. 2007 Aug 21;8:286. doi: 10.1186/1471-2164-8-286 (PMC2031905; doi:10.1186/1471-2164-8-286)
Supplement: Additional file 1 — Complete list and analytical data of the 185 bacterial chromosomes used in this study. The table lists the bacterial chromosomes analyzed in this study and shows all the data obtained from computations, which are used in deriving the arguments and conclusions in this paper. [file 1471-2164-8-286-S1.pdf]

Table S1. Complete list and analytical data of the 185 bacterial chromosomes used in this study

No	Accession	Chromosome	Taxonomy	Group	Length	Putative ori	Putative ter	%G+C	%CDS	χCDS	χG	χG_{nc}	χG_{cd}	σG_d	σG_g	σG^T	σG^R	χA	χA_{nc}	χA_{cd}	σA_d	σA_g	σA^T	σA^R
1	NC_008009	Acidobacteria bacterium Ellin345	Acidobacteria	1	5650368	33	2825217	58.38	89.33	0.086	0.012	0.019	0.012	0.014	-0.010	0.002	0.012	-0.002	0.001	-0.002	0.037	0.048	0.042	-0.006
2	NC_002935	Corynebacterium diphtheriae	Actinobacteria	2	2488635	19	1244336	53.48	87.87	0.272	0.049	0.095	0.043	0.037	-0.054	-0.008	0.045	-0.012	-0.029	-0.009	-0.016	-0.003	-0.009	-0.007
3	NC_004369	Corynebacterium efficiens YS-314	Actinobacteria	2	3147090	1	1573546	63.14	90.52	0.177	0.012	0.052	0.009	-0.022	-0.052	-0.037	0.015	0.001	-0.015	0.003	-0.002	-0.010	-0.006	0.004
4	NC_006958	Corynebacterium glutamicum	Actinobacteria	2	3282708	1	1641355	53.84	87.34	0.174	0.038	0.083	0.032	0.016	-0.055	-0.020	0.035	-0.006	-0.017	-0.004	-0.010	-0.005	-0.007	-0.003
5	NC_002677	Mycobacterium leprae TN	Actinobacteria	2	3268203	1	1634102	57.80	76.64	0.225	0.056	0.056	0.056	0.070	-0.033	0.018	0.051	-0.064	-0.065	-0.063	-0.073	0.048	-0.013	-0.061
6	NC_008146	Mycobacterium sp. MCS	Actinobacteria	2	5705448	20	2852744	68.45	92.92	0.172	0.007	0.022	0.007	0.000	-0.016	-0.008	0.008	0.002	-0.000	0.002	0.012	0.012	0.012	0.000
7	NC_002944	Mycobacterium avium subsp. Paratuberculosis	Actinobacteria	2	4829781	1	2414891	69.30	91.36	0.055	0.005	0.007	0.004	0.003	-0.005	-0.001	0.004	0.001	0.001	0.001	0.011	0.010	0.010	0.001
8	NC_002945	Mycobacterium bovis AF2122/97	Actinobacteria	2	4345492	1	2172747	65.63	90.88	0.177	0.026	0.033	0.026	0.041	-0.005	0.018	0.023	-0.021	-0.018	-0.022	-0.031	0.008	-0.012	-0.019
9	NC_000962	Mycobacterium tuberculosis H37Rv	Actinobacteria	2	4411532	1	2205765	65.61	90.92	0.172	0.026	0.033	0.026	0.040	-0.005	0.017	0.023	-0.022	-0.018	-0.022	-0.031	0.009	-0.011	-0.020
10	NC_006361	Nocardia farcinica IFM 10152	Actinobacteria	2	6021225	1	3010613	70.83	90.30	0.182	0.007	0.030	0.005	-0.011	-0.029	-0.020	0.009	-0.005	-0.023	-0.003	0.001	0.008	0.005	-0.004
11	NC_008268	Rhodococcus sp. RHA1	Actinobacteria	2	7804765	3872441	1	67.52	91.05	0.164	0.019	0.037	0.017	-0.002	-0.043	-0.022	0.021	-0.007	-0.017	-0.006	0.004	0.018	0.011	-0.007
12	NC_007777	Frankia sp. CcI3	Actinobacteria	2	5433628	35	2716849	70.08	84.74	0.139	0.023	0.035	0.021	0.012	-0.033	-0.010	0.022	-0.036	-0.054	-0.032	-0.058	-0.002	-0.030	-0.028
13	NC_004551	Tropheryma whipplei TW08/27	Actinobacteria	2	925938	1	462970	46.31	84.47	0.476	0.019	-0.054	0.033	0.072	0.079	0.076	-0.003	-0.053	-0.009	-0.061	-0.064	0.053	-0.006	-0.059
14	NC_006087	Leifsonia xyli subsp. xyli str. CTCB07	Actinobacteria	2	2584158	225	1292304	67.68	70.10	0.278	0.009	0.012	0.008	-0.012	-0.042	-0.027	0.015	-0.022	-0.028	-0.019	-0.018	0.019	0.001	-0.019
15	NC_006085	Propionibacterium acnes KPA171202	Actinobacteria	2	2560265	245	1280377	60.01	89.18	0.196	0.051	0.085	0.047	0.033	-0.069	-0.018	0.051	-0.048	-0.062	-0.046	-0.075	0.003	-0.036	-0.039
16	NC_003155	Streptomyces avermitilis MA-4680	Actinobacteria	2	9025608	5287935	1	70.72	86.06	0.145	-0.001	0.027	-0.005	-0.026	-0.024	-0.025	-0.001	0.003	-0.012	0.006	0.025	0.020	0.023	0.003
17	NC_003888	Streptomyces coelicolor A3(2)	Actinobacteria	2	8667507	4270314	1	72.12	88.75	0.102	-0.014	0.016	-0.018	-0.046	-0.017	-0.032	-0.015	0.012	0.003	0.013	0.034	0.012	0.023	0.011
18	NC_007333	Thermobifida fusca YX	Actinobacteria	2	3642249	195	1821319	67.50	85.05	0.221	-0.003	0.036	-0.009	-0.038	-0.035	-0.036	-0.001	0.032	0.036	0.032	0.049	-0.004	0.023	0.027
19	NC_004307	Bifidobacterium longum NCC2705	Actinobacteria	2	2256640	1625765	497445	60.12	85.27	0.358	0.000	0.046	-0.007	-0.027	-0.036	-0.032	0.005	0.008	0.002	0.010	0.032	0.038	0.035	-0.003
20	NC_008148	Rubrobacter xylanophilus DSM 9941	Actinobacteria	2	3225748	2	1612876	70.48	91.71	0.226	0.022	0.038	0.021	0.058	0.037	0.047	0.010	-0.014	-0.024	-0.013	-0.012	0.015	0.002	-0.014
21	NC_006177	Symbiobacterium thermophilum IAM 14863	Actinobacteria	2	3566135	1	1783068	68.67	87.00	0.447	0.040	0.075	0.035	0.047	-0.004	0.021	0.025	0.004	0.024	0.001	-0.000	-0.004	-0.002	0.002
22	NC_000918	Aquifex aeolicus VF5	Aquificae	3	1551335	209841	985508	43.48	92.99	0.004	-0.000	-0.012	0.001	0.115	0.115	0.115	0.000	-0.008	-0.003	-0.008	0.147	0.166	0.157	-0.009
23	NC_002950	Porphyromonas gingivalis W83	Bacteroidetes	4	2343476	1	1171739	48.29	82.43	0.085	0.018	0.026	0.017	0.041	0.011	0.026	0.015	-0.010	-0.010	-0.010	0.028	0.055	0.041	-0.013
24	NC_004663	Bacteroides thetaiotaomicron VPI-5482	Bacteroidetes	4	6260361	4041000	910819	42.84	89.48	0.165	0.070	0.076	0.070	0.129	0.009	0.069	0.060	-0.031	-0.029	-0.031	0.023	0.108	0.066	-0.043
25	NC_002932	Chlorobium tepidum TLS	Chlorobi	5	2154946	2879	1080352	56.53	87.86	0.117	0.039	0.063	0.036	0.028	-0.047	-0.010	0.037	-0.043	-0.050	-0.043	-0.001	0.096	0.048	-0.048
26	NC_007514	Chlorobium chlorochromatii CaD3	Chlorobi	5	2572079	346	1286385	44.28	88.28	0.227	0.042	0.039	0.043	0.081	0.019	0.050	0.031	-0.019	-0.030	-0.017	-0.017	0.017	0.000	-0.017
27	NC_007512	Pelodictyon luteolum DSM 273	Chlorobi	5	2364842	2363373	1180952	57.33	89.01	0.160	0.038	0.058	0.036	0.009	-0.074	-0.033	0.041	-0.058	-0.075	-0.056	-0.012	0.117	0.053	-0.064
28	NC_007356	Dehalococcoides sp. CBDB1	Chloroflexi	6	1395502	261	698012	47.03	89.89	0.035	0.048	0.084	0.044	0.084	-0.003	0.041	0.043	-0.035	-0.018	-0.037	-0.001	0.077	0.038	-0.039
29	NC_007775	Synechococcus sp. JA-3-3Ab	Cyanobacteria	7	2932766	71	1466454	60.24	84.37	-0.024	0.000	-0.002	0.001	0.009	0.007	0.008	0.001	0.001	-0.003	0.002	-0.032	-0.034	-0.033	0.001
30	NC_000911	Synechocystis sp. PCC 6803	Cyanobacteria	7	3573470	349000	2135735	47.72	86.85	-0.017	0.001	0.003	0.001	0.027	0.024	0.026	0.001	-0.000	0.003	-0.001	-0.005	-0.003	-0.004	-0.001
31	NC_004113	Thermosynechococcus elongatus BP-1	Cyanobacteria	7	2593857	2266000	969071	53.92	89.51	-0.016	0.003	0.001	0.003	-0.003	-0.009	-0.006	0.003	0.001	-0.002	0.001	-0.036	-0.037	-0.037	0.001
32	NC_005125	Gloeobacter violaceus PCC 7421	Cyanobacteria	7	4659019	1585087	3914596	62.00	89.15	-0.003	0.001	0.002	0.001	-0.010	-0.012	-0.011	0.001	0.001	0.002	0.000	-0.012	-0.012	-0.012	0.000
33	NC_003272	Nostoc sp. PCC 7120	Cyanobacteria	7	6413771	2403018	5609903	41.35	82.15	0.014	0.001	0.000	0.001	0.036	0.036	0.036	0.000	-0.001	-0.001	-0.001	0.028	0.032	0.030	-0.002
34	NC_008025	Deinococcus geothermalis DSM 11300	Deinococcus-Thermus	8	2467205	222	1233824	66.64	90.26	0.003	0.008	0.028	0.006	0.015	0.003	0.009	0.006	-0.024	-0.017	-0.024	-0.018	0.030	0.006	-0.024
35	NC_001263	Deinococcus radiodurans R1 # 1	Deinococcus-Thermus	8	2648638	3268	1327587	67.01	89.75	0.029	0.001	0.019	-0.001	-0.013	-0.011	-0.012	-0.001	-0.010	-0.005	-0.011	0.027	0.052	0.040	-0.013
36	NC_006461	Thermus thermophilus HB8	Deinococcus-Thermus	8	1849742	1848185	923314	69.52	95.16	0.016	0.015	0.025	0.015	0.004	-0.026	-0.011	0.015	-0.017	-0.010	-0.017	-0.042	-0.008	-0.025	-0.017
37	NC_006582	Bacillus clausii KSM-K16	Firmicutes; Bacillales	9	4303871	183	2152118	44.75	85.77	0.529	0.080	0.109	0.077	0.105	0.016	0.061	0.044	0.030	0.036	0.029	0.051	0.041	0.046	0.005
38	NC_002570	Bacillus halodurans C-125	Firmicutes; Bacillales	9	4202352	584	2101760	43.69	85.03	0.535	0.095	0.118	0.092	0.130	0.037	0.083	0.046	0.030	0.033	0.030	0.053	0.044	0.048	0.004
39	NC_006270	Bacillus licheniformis ATCC 14580	Firmicutes; Bacillales	9	4222334	507	2111674	46.20	85.89	0.513	0.065	0.095	0.061	0.088	0.023	0.056	0.032	0.048	0.029	0.051	0.093	0.078	0.085	0.008
40	NC_000964	Bacillus subtilis subsp. subtilis str. 168	Firmicutes; Bacillales	9	4214630	410	2107725	43.52	87.07	0.496	0.075	0.097	0.072	0.107	0.030	0.068	0.038	0.041	0.030	0.043	0.081	0.067	0.074	0.007
41	NC_003997	Bacillus anthracis str. Ames	Firmicutes; Bacillales	9	5227293	407	2614053	35.38	80.61	0.507	0.157	0.182	0.152	0.208	0.022	0.115	0.093	0.047	0.035	0.050	0.086	0.057	0.071	0.014
42	NC_004722	Bacillus cereus ATCC 14579	Firmicutes; Bacillales	9	5411809	281	2706185	35.28	80.65	0.530	0.163	0.184	0.159	0.210	0.010	0.110	0.100	0.051	0.034	0.055	0.089	0.055	0.072	0.017
43	NC_005957	Bacillus thuringiensis serovar konkukian str. 97-27	Firmicutes; Bacillales	9	5237682	409	2619250	35.41	83.78	0.506	0.159	0.191	0.154	0.208	0.015	0.112	0.096	0.048	0.031	0.051	0.086	0.055	0.071	0.015
44	NC_006510	Geobacillus kaustophilus HTA426	Firmicutes; Bacillales	9	3544776	88	1772476	52.09	84.85	0.632	0.090	0.122	0.086	0.105	-0.000	0.052	0.053	0.021	0.033	0.019	0.036	0.053	0.044	-0.009
45	NC_004193	Oceanobacillus iheyensis HTE831	Firmicutes; Bacillales	9	3630528	300	1815564	35.68	84.23	0.536	0.119	0.125	0.118	0.174	0.070	0.122	0.052	0.044	0.030	0.047	0.081	0.065	0.073	0.008
46	NC_003212	Listeria innocua Clip11262	Firmicutes; Bacillales	9	3011208	319	1505923	37.44	89.05	0.603	0.096	0.150	0.091	0.122	0.036	0.079	0.043	0.063	0.045	0.066	0.094	0.048	0.071	0.023
47	NC_003210	Listeria monocytogenes EGD-e	Firmicutes; Bacillales	9	2944528	318	1472582	37.98	89.11	0.588	0.097	0.152	0.092	0.123	0.030	0.076	0.047	0.058	0.038	0.061	0.088	0.045	0.067	0.021
48	NC_002758	Staphylococcus aureus subsp. aureus Mu50	Firmicutes; Bacillales	9	2878529	517	1439537	32.88	83.66	0.536	0.116	0.147	0.111	0.154	0.035	0.094	0.060	0.054	0.036	0.058	0.094	0.060	0.077	0.017
49	NC_004461	Staphylococcus epidermidis ATCC 12228	Firmicutes; Bacillales	9	2499279	362	1250001	32.10	83.31	0.440	0.098	0.125	0.093	0.142	0.035	0.089	0.053	0.036	0.019	0.040	0.085	0.077	0.081	0.004
50	NC_007168	Staphylococcus haemolyticus JCSC1435	Firmicutes; Bacillales	9	2685015	508	1343015	32.79	85.87	0.400	0.083	0.113	0.079	0.135	0.055	0.095	0.040	0.038	0.025	0.041	0.089	0.071	0.080	0.009
51	NC_007350	Staphylococcus saprophyticus subsp. Saprophyticus	Firmicutes; Bacillales	9	2516575	509	1258796	33.24	83.60	0.448	0.112	0.152	0.105	0.156	0.029	0.093	0.063	0.043	0.031	0.046	0.085	0.058	0.072	0.014
52	NC_003030	Clostridium acetobutylicum ATCC 824	Firmicutes; Clostridia	10	3940880	467	1970907	30.93	85.69	0.584	0.210	0.225	0.208	0.278	0.059	0.169	0.110	0.077	0.058	0.080	0.123	0.085	0.104	0.019
53	NC_003366	Clostridium perfringens str. 13	Firmicutes; Clostridia	10	3031430	410	1516125	28.57	83.40	0.616	0.223	0.236	0.221	0.308	0.153	0.231	0.078	0.082	0.042	0.091	0.132	0.081	0.107	0.026
54	NC_004557	Clostridium tetani E88	Firmicutes; Clostridia	10	2799251	52253	1451878	28.75	85.87	0.628	0.213	0.205	0.215	0.289	0.114	0.201	0.087	0.115	0.077	0.121	0.165	0.067	0.116	0.049
55	NC_007907	Desulfitobacterium hafniense Y51	Firmicutes; Clostridia	10	5727534	1	2863768	47.36	84.69	0.367	0.054	0.079	0.050	0.089	0.035	0.062	0.027	0.014	0.028	0.011	0.024	0.017	0.020	0.003
56	NC_007644	Moorella thermoacetica ATCC 39073	Firmicutes; Clostridia	10	2628784	450	1314842	55.79	86.20	0.626	0.043	0.090	0.037	0.041	-0.017	0.012	0.029	0.015	0.033	0.012	0.020	0.021	0.021	-0.001
57	NC_003869	Thermoanaerobacter tengcongensis MB4	Firmicutes; Clostridia	10	2689445	352	1345074	37.57	86.91	0.737	0.193	0.183	0.195	0.235	0.083	0.159	0.076	0.078	0.056	0.081	0.116	0.145	0.131	-0.015
58	NC_004668	Enterococcus faecalis V583	Firmicutes; Lactobacillales	11	3218031	59	1609074	37.53	86.01	0.631	0.105	0.133	0.101	0.127	0.019	0.073	0.054	0.055	0.054	0.055	0.077	0.039	0.058	0.019
59	NC_006814	Lactobacillus acidophilus NCFM	Firmicutes; Lactobacillales	11	1993564	31	996813	34.71	88.11	0.503	0.108	0.130	0.106	0.151	0.031	0.091	0.060	0.019	0.027	0.018	0.038	0.042	0.040	-0.002
60	NC_008054	Lactobacillus delbrueckii subsp. Bulgaricus	Firmicutes; Lactobacillales	11	1864998	323	932822	49.72	73.29	0.577	0.049	0.076	0.041	0.036	-0.058	-0.011	0.047	0.046	0.039	0.049	0.079	0.063	0.071	0.008
61	NC_005362	Lactobacillus johnsonii NCC 533	Firmicutes; Lactobacillales	11	1992676	1	996339	34.61	89.19	0.499	0.108	0.153	0.103	0.147	0.028	0.088	0.059	0.030	0.046	0.028	0.049	0.033	0.041	0.008
62	NC_004567	Lactobacillus plantarum WCFS1	Firmicutes; Lactobacillales	11	3308274	1	1654138	44.47	84.41	0.511	0.107	0.151	0.100	0.108	-0.075	0.017	0.092	-0.001	0.002	-0.002	0.008	0.032	0.020	-0.012
63	NC_007576	Lactobacillus sakei subsp. sakei 23K	Firmicutes; Lactobacillales	11	1884661	210	942540	41.26	86.77	0.593	0.095	0.151	0.088	0.086	-0.094	-0.004	0.090	0.031	0.032	0.032	0.049	0.039	0.044	0.005
64	NC_007929	Lactobacillus salivarius subsp. Salivarius	Firmicutes; Lactobacillales	11	1827111	1	913556	32.94	84.06	0.656	0.167	0.183	0.165	0.204	0.025	0.115	0.090	0.059	0.042	0.062	0.078	0.010	0.044	0.034
65	NC_002662	Lactococcus lactis subsp. lactis Il1403	Firmicutes; Lactobacillales	11	2365589	358	1183152	35.33	85.31	0.614	0.092	0.123	0.087	0.114	0.024	0.069	0.045	0.033	0.050	0.030	0.042	0.020	0.031	0.011
66	NC_004116	Streptococcus agalactiae 2603V/R	Firmicutes; Lactobacillales	11	2160267	102	1080235	35.65	86.40	0.560	0.105	0.141	0.100	0.129	0.005	0.067	0.062	0.014	0.028	0.012	0.029	0.047	0.038	-0.009
67	NC_004350	Streptococcus mutans UA159	Firmicutes; Lactobacillales	11	2030921	194	1015654	36.83	85.70	0.638	0.101	0.134	0.097	0.118	0.001	0.060	0.059	-0.003	0.010	-0.005	-0.005	0.006	0.000	-0.005

Table S1. Complete list and analytical data of the 185 bacterial chromosomes used in this study

68	NC_003098	Streptococcus pneumoniae R6	Firmicutes; Lactobacillales	11	2038615	1	1019308	39.72	86.47	0.627	0.099	0.126	0.096	0.109	-0.042	0.033	0.075	0.010	0.028	0.008	0.019	0.041	0.030	-0.011
69	NC_002737	Streptococcus pyogenes M1 GAS	Firmicutes; Lactobacillales	11	1852441	232	926452	38.51	83.63	0.578	0.086	0.122	0.080	0.095	-0.021	0.037	0.058	0.018	0.035	0.014	0.026	0.030	0.028	-0.002
70	NC_006449	Streptococcus thermophilus CNRZ1066	Firmicutes; Lactobacillales	11	1796226	186	898299	39.08	83.67	0.619	0.092	0.125	0.086	0.100	-0.028	0.036	0.064	-0.007	0.013	-0.011	-0.006	0.030	0.012	-0.018
71	NC_007716	Aster yellows witches'-broom phytoplasma AYWB	Firmicutes; Mollicutes	12	706569	1	353285	26.89	73.66	0.411	0.004	-0.003	0.007	-0.031	-0.102	-0.067	0.035	0.007	-0.001	0.010	0.079	0.153	0.116	-0.037
72	NC_005303	Onion yellows phytoplasma OY-M	Firmicutes; Mollicutes	12	860631	1	430316	27.74	73.00	0.356	0.001	-0.010	0.005	-0.039	-0.098	-0.069	0.029	0.012	0.002	0.016	0.087	0.134	0.110	-0.023
73	NC_007633	Mycoplasma capricolum subsp. Capricolum	Firmicutes; Mollicutes	12	1010023	1	505012	23.77	88.24	0.401	0.071	0.059	0.072	0.159	0.130	0.145	0.015	0.030	0.013	0.032	0.092	0.108	0.100	-0.008
74	NC_004829	Mycoplasma gallisepticum R	Firmicutes; Mollicutes	12	996422	3163	501374	31.45	87.27	0.629	0.032	0.053	0.029	0.046	0.045	0.045	0.000	0.052	0.016	0.057	0.089	0.080	0.085	0.005
75	NC_000908	Mycoplasma genitalium G-37	Firmicutes; Mollicutes	12	580074	578581	288544	31.69	90.79	0.670	0.042	0.028	0.044	0.064	0.064	0.064	0.000	0.031	0.006	0.034	0.056	0.073	0.064	-0.009
76	NC_006360	Mycoplasma hyopneumoniae 232	Firmicutes; Mollicutes	12	892758	1	446380	28.56	89.76	0.108	0.002	0.024	-0.000	0.032	0.042	0.037	-0.005	0.013	-0.001	0.014	0.087	0.075	0.081	0.006
77	NC_006908	Mycoplasma mobile 163K	Firmicutes; Mollicutes	12	777079	215	388754	24.95	90.64	0.277	0.010	-0.002	0.012	0.111	0.170	0.140	-0.029	0.018	0.024	0.018	0.085	0.099	0.092	-0.007
78	NC_004432	Mycoplasma penetrans HF-2	Firmicutes; Mollicutes	12	1358633	1	679317	25.72	88.60	0.717	0.089	0.079	0.090	0.120	0.105	0.112	0.007	0.045	-0.019	0.054	0.084	0.120	0.102	-0.018
79	NC_000912	Mycoplasma pneumoniae M129	Firmicutes; Mollicutes	12	816394	814787	406590	40.01	87.82	0.626	0.015	0.010	0.016	0.022	0.012	0.017	0.005	0.030	0.002	0.035	0.062	0.082	0.072	-0.010
80	NC_002771	Mycoplasma pulmonis UAB CTIP	Firmicutes; Mollicutes	12	963879	222	482161	26.64	90.03	0.208	0.023	0.035	0.023	0.071	0.051	0.061	0.010	0.020	0.015	0.022	0.109	0.110	0.109	-0.001
81	NC_002162	Ureaplasma parvum serovar 3 str.	Firmicutes; Mollicutes	12	751719	1	375860	25.50	91.14	0.326	0.041	0.081	0.037	0.105	0.099	0.102	0.003	0.024	0.001	0.027	0.078	0.075	0.077	0.002
82	NC_003454	Fusobacterium nucleatum subsp. nucleatum	Fusobacteria	13	2174500	641868	1729118	27.15	88.86	0.271	0.102	0.134	0.098	0.284	0.230	0.257	0.027	0.053	0.051	0.053	0.137	0.094	0.115	0.022
83	NC_002696	Caulobacter crescentus CB15	Proteobacteria; α-	14	4016947	5636	2014109	67.21	90.25	0.100	0.015	0.024	0.015	0.000	-0.032	-0.016	0.016	-0.014	-0.022	-0.012	-0.010	0.016	0.003	-0.013
84	NC_004463	Bradyrhizobium japonicum USDA 110	Proteobacteria; α-	14	9105828	895124	5448038	64.06	86.67	0.119	0.010	0.016	0.010	-0.005	-0.028	-0.016	0.012	-0.011	-0.010	-0.011	-0.004	0.020	0.008	-0.012
85	NC_007964	Nitrobacter hamburgensis X14	Proteobacteria; α-	14	4406967	107	2203590	61.71	80.39	0.132	0.018	0.019	0.018	0.020	-0.016	0.002	0.018	-0.014	-0.009	-0.016	-0.003	0.033	0.015	-0.018
86	NC_007406	Nitrobacter winogradskyi Nb-255	Proteobacteria; α-	14	3402093	567	1701613	62.05	83.98	0.124	0.022	0.026	0.021	0.026	-0.015	0.005	0.021	-0.019	-0.017	-0.019	-0.011	0.030	0.010	-0.021
87	NC_006932	Brucella abortus biovar 1 str. 9-941 # I	Proteobacteria; α-	14	2124241	784	1062904	57.16	81.00	0.180	0.032	0.038	0.031	0.038	-0.021	0.009	0.030	-0.022	-0.022	-0.022	-0.034	0.004	-0.015	-0.019
88	NC_003317	Brucella melitensis 16M # I	Proteobacteria; α-	14	2117144	2000	1060572	57.16	85.41	0.230	0.034	0.046	0.032	0.038	-0.023	0.007	0.030	-0.024	-0.019	-0.025	-0.036	0.007	-0.014	-0.022
89	NC_003318	Brucella melitensis 16M # II	Proteobacteria; α-	14	1177787	117000	705893	57.34	87.47	0.073	0.032	0.051	0.030	0.038	-0.020	0.009	0.029	-0.027	-0.025	-0.028	-0.058	-0.008	-0.033	-0.025
90	NC_004310	Brucella suis 1330 # I	Proteobacteria; α-	14	2107794	2094000	1040104	57.21	84.27	0.171	0.033	0.045	0.031	0.038	-0.021	0.009	0.029	-0.023	-0.022	-0.023	-0.038	0.003	-0.018	-0.020
91	NC_004311	Brucella suis 1330 # II	Proteobacteria; α-	14	1207381	1181000	577309	57.32	84.20	0.061	0.032	0.042	0.031	0.039	-0.022	0.009	0.030	-0.027	-0.020	-0.029	-0.059	-0.005	-0.032	-0.027
92	NC_008254	Mesorhizobium sp. BNC1	Proteobacteria; α-	14	4412446	85	2206308	61.07	89.34	0.007	0.008	0.011	0.007	0.019	0.004	0.012	0.007	-0.006	-0.013	-0.005	-0.016	-0.007	-0.012	-0.005
93	NC_003062	Agrobacterium tumefaciens str. C58 circular	Proteobacteria; α-	14	2841581	2800000	1379209	59.38	88.91	0.108	0.024	0.042	0.022	0.013	-0.034	-0.010	0.023	-0.023	-0.033	-0.021	-0.035	0.004	-0.015	-0.020
94	NC_007761	Rhizobium etli CFN 42	Proteobacteria; α-	14	4381608	370494	2561298	61.27	86.03	0.079	0.010	0.019	0.008	-0.014	-0.034	-0.024	0.010	-0.006	-0.010	-0.005	-0.006	0.004	-0.001	-0.005
95	NC_003047	Sinorhizobium meliloti 1021	Proteobacteria; α-	14	3654135	3289000	1461932	62.73	85.80	0.023	0.013	0.019	0.012	-0.001	-0.025	-0.013	0.012	-0.008	-0.008	-0.008	-0.001	0.016	0.008	-0.008
96	NC_007493	Rhodobacter sphaeroides 2.4.1 # 1	Proteobacteria; α-	14	3188609	3110775	1516470	69.01	88.97	0.093	0.016	0.027	0.015	0.019	-0.010	0.005	0.014	-0.007	-0.005	-0.007	-0.029	-0.020	-0.025	-0.005
97	NC_008209	Roseobacter denitrificans OCh 114	Proteobacteria; α-	14	4133097	204958	2271506	58.97	89.43	0.084	0.028	0.033	0.027	0.066	0.019	0.043	0.024	-0.014	-0.012	-0.014	-0.027	-0.001	-0.014	-0.013
98	NC_003911	Silicibacter pomeroyi DSS-3	Proteobacteria; α-	14	4109442	164331	2219052	64.22	89.51	0.061	0.022	0.034	0.021	0.046	0.007	0.027	0.019	-0.007	-0.013	-0.006	-0.023	-0.013	-0.018	-0.005
99	NC_006677	Gluconobacter oxydans 621H	Proteobacteria; α-	14	2702173	155	1351241	61.07	89.81	-0.060	-0.037	-0.044	-0.037	-0.040	0.034	-0.003	-0.037	0.018	0.013	0.018	-0.012	-0.045	-0.029	0.016
100	NC_007643	Rhodospirillum rubrum ATCC 11170	Proteobacteria; α-	14	4352825	228	2176640	65.45	88.28	0.040	0.026	0.045	0.024	0.002	-0.047	-0.023	0.025	-0.019	-0.013	-0.019	-0.078	-0.044	-0.061	-0.017
101	NC_007940	Rickettsia bellii RML369-C	Proteobacteria; α-	14	1522076	934199	173161	31.65	84.96	-0.048	-0.021	-0.017	-0.021	0.109	0.139	0.124	-0.015	-0.000	0.009	-0.002	0.082	0.078	0.080	0.002
102	NC_003103	Rickettsia conorii str. Malish 7	Proteobacteria; α-	14	1268755	1	634378	32.44	80.32	0.216	0.064	0.043	0.068	0.148	0.058	0.103	0.045	0.007	0.003	0.008	0.065	0.079	0.072	-0.007
103	NC_000963	Rickettsia prowazekii str. Madrid E	Proteobacteria; α-	14	1111523	1	555762	29.00	75.48	0.235	0.073	0.066	0.077	0.166	0.071	0.118	0.048	-0.009	-0.033	0.001	0.048	0.074	0.061	-0.013
104	NC_002978	Wolbachia endosymbiont of D. melanogaster	Proteobacteria; α-	14	1267782	153	634044	35.23	80.05	-0.062	-0.003	-0.000	-0.004	0.162	0.151	0.157	0.006	-0.009	-0.001	-0.010	0.090	0.099	0.094	-0.005
105	NC_006833	Wolbachia endosymbiont of Brugia malayi	Proteobacteria; α-	14	1080084	361157	901199	34.18	66.99	-0.152	-0.036	-0.026	-0.041	0.140	0.172	0.156	-0.016	0.002	0.005	0.001	0.099	0.072	0.085	0.014
106	NC_007205	Candidatus Pelagibacter ubique HTCC1062	Proteobacteria; α-	14	1308759	398915	1053294	29.68	95.53	0.281	0.053	0.024	0.054	0.161	0.142	0.152	0.010	0.023	0.009	0.024	0.093	0.098	0.095	-0.003
107	NC_007794	Novosphingobium aromaticivorans DSM 12444	Proteobacteria; α-	14	3561584	24	1780816	65.15	91.48	0.113	0.015	0.028	0.014	0.004	-0.027	-0.012	0.015	-0.014	-0.010	-0.014	-0.006	0.025	0.009	-0.015
108	NC_008048	Sphingopyxis alaskensis RB2256	Proteobacteria; α-	14	3345170	173	1672758	65.50	90.39	0.018	0.012	0.014	0.012	0.017	-0.007	0.005	0.012	-0.007	-0.010	-0.007	-0.004	0.009	0.003	-0.007
109	NC_002927	Bordetella bronchiseptica RB50	Proteobacteria; β	15	5339179	5311567	2641977	68.08	91.81	0.117	0.029	0.047	0.028	0.016	-0.043	-0.013	0.030	-0.021	-0.027	-0.020	-0.008	0.036	0.014	-0.022
110	NC_002928	Bordetella parapertussis 12822	Proteobacteria; β	15	4773551	4745937	2359161	68.10	86.52	0.110	0.025	0.033	0.024	0.012	-0.039	-0.014	0.026	-0.015	-0.028	-0.012	-0.004	0.023	0.010	-0.013
111	NC_002929	Bordetella pertussis Tohama I	Proteobacteria; β	15	4086189	69000	2112094	67.72	82.59	0.119	0.016	0.017	0.015	-0.002	-0.038	-0.020	0.018	-0.009	-0.013	-0.008	0.006	0.025	0.016	-0.010
112	NC_007951	Burkholderia xenovorans LB400 # 1	Proteobacteria; β	15	4895836	192	2448110	62.76	85.91	0.174	0.026	0.033	0.024	0.035	-0.010	0.013	0.022	-0.006	-0.004	-0.006	0.005	0.022	0.013	-0.009
113	NC_007510	Burkholderia sp. 383 # 1	Proteobacteria; β	15	3694126	76	1847139	66.21	87.70	0.175	0.031	0.048	0.029	0.030	-0.027	0.001	0.029	-0.008	-0.006	-0.009	0.009	0.034	0.021	-0.013
114	NC_007434	Burkholderia pseudomallei 1710b # I	Proteobacteria; β	15	4126292	312239	2375385	67.61	85.52	0.085	0.033	0.053	0.029	0.033	-0.024	0.005	0.028	-0.009	-0.017	-0.006	0.000	0.013	0.007	-0.007
115	NC_007651	Burkholderia thailandensis E264 # I	Proteobacteria; β	15	3809201	3690347	1785746	67.29	86.60	0.081	0.016	0.032	0.014	0.025	-0.002	0.011	0.013	-0.010	-0.007	-0.011	-0.001	0.023	0.011	-0.012
116	NC_007347	Ralstonia eutropha JMP134 # 1	Proteobacteria; β	15	3806533	184	1903450	64.68	88.84	0.194	0.021	0.032	0.019	0.021	-0.017	0.002	0.019	-0.003	-0.008	-0.002	0.011	0.023	0.017	-0.006
117	NC_003295	Ralstonia solanacearum GMI1000	Proteobacteria; β	15	3716413	3716276	1858069	67.04	87.87	0.216	0.022	0.037	0.020	0.012	-0.033	-0.011	0.023	-0.011	-0.030	-0.008	0.015	0.045	0.030	-0.015
118	NC_007404	Thiobacillus denitrificans ATCC 25259	Proteobacteria; β	15	2909809	137	1455041	66.07	92.34	0.189	0.016	0.034	0.014	-0.013	-0.054	-0.034	0.020	-0.002	0.003	-0.002	0.023	0.041	0.032	-0.009
119	NC_005085	Chromobacterium violaceum ATCC 12472	Proteobacteria; β	15	4751080	190000	2565540	64.83	88.47	0.149	0.046	0.083	0.042	0.042	-0.042	0.000	0.042	-0.028	-0.044	-0.025	0.015	0.079	0.047	-0.032
120	NC_002946	Neisseria gonorrhoeae FA 1090	Proteobacteria; β	15	2153922	159	1077120	52.69	78.12	0.033	0.026	0.035	0.024	0.010	-0.038	-0.014	0.024	-0.004	0.011	-0.009	0.064	0.088	0.076	-0.012
121	NC_003112	Neisseria meningitidis MC58	Proteobacteria; β	15	2272351	6000	1142175	51.53	79.37	0.069	0.051	0.061	0.049	0.031	-0.069	-0.019	0.050	-0.019	-0.010	-0.021	0.052	0.107	0.080	-0.028
122	NC_003116	Neisseria meningitidis Z2491	Proteobacteria; β	15	2184406	240000	1332203	51.81	80.64	0.065	0.056	0.071	0.054	0.037	-0.073	-0.018	0.055	-0.024	-0.015	-0.026	0.045	0.109	0.077	-0.032
123	NC_004757	Nitrosomonas europaea ATCC 19718	Proteobacteria; β	15	2812094	211	1406258	50.72	88.06	0.102	0.045	0.049	0.044	0.079	-0.002	0.038	0.041	-0.023	-0.028	-0.022	-0.007	0.042	0.018	-0.024
124	NC_007614	Nitrosospira multiformis ATCC 25196 # 1	Proteobacteria; β	15	3184243	69	1592190	53.94	85.36	0.202	0.029	0.036	0.028	0.061	0.023	0.042	0.019	-0.002	-0.005	-0.002	0.017	0.030	0.023	-0.007
125	NC_006513	Azoarcus sp. EbN1	Proteobacteria; β	15	4296230	1693960	3842075	65.12	90.81	0.131	0.021	0.038	0.020	0.016	-0.025	-0.005	0.021	-0.010	-0.005	-0.011	0.007	0.034	0.021	-0.013
126	NC_007298	Dechloromonas aromatica RCB	Proteobacteria; β	15	4501104	151	2250703	59.25	91.53	0.112	0.048	0.069	0.047	0.030	-0.067	-0.018	0.048	-0.038	-0.044	-0.038	-0.031	0.046	0.008	-0.039
127	NC_005363	Bdellovibrio bacteriovorus HD100	Proteobacteria; δ-	16	3782950	1	1891476	50.65	92.85	0.120	0.058	0.071	0.057	0.083	-0.025	0.029	0.054	-0.023	-0.029	-0.022	0.000	0.050	0.025	-0.025
128	NC_002939	Geobacter sulfurreducens PCA	Proteobacteria; δ-	16	3814139	189000	2096069	60.94	89.52	0.228	0.017	0.045	0.014	0.005	-0.028	-0.011	0.017	-0.018	-0.019	-0.018	0.004	0.054	0.029	-0.025
129	NC_007498	Pelobacter carbinolicus DSM 2380	Proteobacteria; δ-	16	3665893	72	1833018	55.11	85.85	0.257	0.064	0.093	0.060	0.063	-0.054	0.004	0.059	-0.052	-0.052	-0.052	-0.051	0.053	0.001	-0.052
130	NC_008095	Myxococcus xanthus DK 1622	Proteobacteria; δ-	16	9139763	87	4569968	68.89	90.52	0.089	0.037	0.052	0.035	0.053	-0.014	0.020	0.033	-0.027	-0.041	-0.025	-0.026	0.024	-0.001	-0.025
131	NC_007759	Syntrophus aciditrophicus SB	Proteobacteria; δ-	16	3179300	155	1589805	51.46	88.45	0.119	0.036	0.045	0.035	0.071	0.010	0.040	0.030	-0.001	-0.001	-0.001	0.031	0.042	0.037	-0.005
132	NC_002163	Campylobacter jejuni subsp. Jejuni	Proteobacteria; ε-	17	1641481	1	820741	30.55	92.79	0.267	0.111	0.107	0.111	0.221	0.083	0.152	0.069	-0.005	-0.003	-0.004	0.035	0.073	0.054	-0.019
133	NC_007575	Thiomicrospira denitrificans ATCC 33889	Proteobacteria; ε-	17	2201561	15	1100795	34.46	92.71	0.214	0.061	0.063	0.061	0.159	0.091	0.125	0.034	0.013	0.015	0.013	0.081	0.093	0.087	-0.006
134	NC_008229	Helicobacter acinonychis str. Sheeba	Proteobacteria; ε-	17	1553927	1	776964	38.18	89.57	0.170	0.055	0.066	0.054	0.135	0.060	0.098	0.038	0.002	-0.004	0.003	0.048	0.061	0.054	-0.007
135	NC_004917	Helicobacter hepaticus ATCC 51449	Proteobacteria; ε-	17	1799146	1081989	182416	35.93	93.05	0.093	0.056	0.067	0.055	0.143	0.051	0.097	0.046	-0.007	0.003	-0.008	0.024	0.047	0.035	-0.011

136	NC_000915	Helicobacter pylori 26695	Proteobacteria; ε-	17	1667867	1608997	775063	38.87	90.20	0.135	0.045	0.052	0.044	0.120	0.055	0.088	0.032	0.009	0.016	0.008	0.062	0.063	0.063	-0.001
137	NC_000921	Helicobacter pylori J99	Proteobacteria; ε-	17	1643831	1559162	737246	39.19	90.21	0.133	0.044	0.059	0.043	0.118	0.055	0.087	0.031	0.006	0.002	0.006	0.060	0.065	0.063	-0.002
138	NC_005090	Wolinella succinogenes DSM 1740	Proteobacteria; ε-	17	2110355	1	1055178	48.46	93.70	0.160	0.080	0.083	0.080	0.103	-0.048	0.027	0.076	-0.014	0.012	-0.016	0.006	0.048	0.027	-0.021
139	NC_007912	Saccharophagus degradans 2-40	Proteobacteria; γ-	18	5057531	5018983	2490217	45.83	86.67	0.267	0.079	0.093	0.077	0.094	-0.048	0.023	0.071	-0.033	-0.062	-0.028	0.016	0.103	0.059	-0.044
140	NC_003910	Colwellia psychrerythraea 34H	Proteobacteria; γ-	18	5373180	72	2686662	38.01	84.48	0.197	0.046	0.053	0.045	0.100	0.038	0.069	0.031	-0.007	-0.013	-0.006	0.014	0.036	0.025	-0.011
141	NC_006512	Idiomarina loihiensis L2TR	Proteobacteria; γ-	18	2839318	362	1420021	47.04	92.02	0.135	0.028	0.023	0.029	0.090	0.052	0.071	0.019	-0.002	0.001	-0.002	0.013	0.023	0.018	-0.005
142	NC_004347	Shewanella oneidensis MR-1	Proteobacteria; γ-	18	4969803	6873	2491774	45.96	83.26	0.116	0.057	0.071	0.054	0.082	-0.018	0.032	0.050	-0.032	-0.039	-0.031	-0.015	0.050	0.018	-0.033
143	NC_002528	Buchnera aphidicola str. APS	Proteobacteria; γ-	18	640681	13918	334258	26.31	86.93	0.101	0.036	0.062	0.034	0.127	0.084	0.105	0.022	-0.031	-0.042	-0.030	0.038	0.113	0.075	-0.037
144	NC_000913	Escherichia coli K12	Proteobacteria; γ-	18	4639675	3881357	1561746	50.79	86.87	0.097	0.032	0.050	0.029	0.076	0.028	0.052	0.024	-0.004	-0.002	-0.004	-0.003	0.005	0.001	-0.004
145	NC_002695	Escherichia coli O157:H7 str. Sakai	Proteobacteria; γ-	18	5498450	4723921	1974696	50.54	85.97	0.169	0.039	0.050	0.037	0.083	0.028	0.055	0.027	-0.002	-0.008	-0.001	0.010	0.016	0.013	-0.003
146	NC_004547	Erwinia carotovora subsp. atroseptica SCRI1043	Proteobacteria; γ-	18	5064019	4985096	2453086	50.97	85.88	0.123	0.055	0.065	0.053	0.094	-0.002	0.046	0.048	-0.022	-0.031	-0.020	-0.018	0.023	0.002	-0.021
147	NC_005126	Photorhabdus luminescens subsp. laumondii TTO1	Proteobacteria; γ-	18	5688987	223	2844716	42.83	83.57	0.184	0.077	0.093	0.075	0.128	0.004	0.066	0.062	-0.029	-0.037	-0.027	-0.012	0.049	0.019	-0.031
148	NC_003198	Salmonella enterica subsp. enterica serovar Typhi	Proteobacteria; γ-	18	4809037	3805127	1400608	52.09	87.47	0.159	0.041	0.053	0.039	0.082	0.020	0.051	0.031	-0.008	-0.012	-0.007	-0.007	0.008	0.001	-0.007
149	NC_003197	Salmonella typhimurium LT2	Proteobacteria; γ-	18	4857432	4045024	1616308	52.22	86.26	0.170	0.043	0.053	0.042	0.083	0.016	0.049	0.033	-0.007	-0.012	-0.007	-0.006	0.007	0.000	-0.007
150	NC_007613	Shigella boydii Sb227	Proteobacteria; γ-	18	4519823	3689193	1429281	51.21	80.33	0.073	0.015	0.020	0.014	0.066	0.047	0.057	0.010	-0.001	0.002	-0.001	0.006	0.010	0.008	-0.002
151	NC_007606	Shigella dysenteriae Sd197	Proteobacteria; γ-	18	4369232	3919608	1734992	51.25	77.02	0.039	0.014	0.020	0.012	0.062	0.042	0.052	0.010	-0.003	-0.003	-0.002	0.008	0.014	0.011	-0.003
152	NC_004337	Shigella flexneri 2a str. 301	Proteobacteria; γ-	18	4607203	3869099	1565497	50.89	86.54	0.100	0.027	0.039	0.025	0.072	0.032	0.052	0.020	-0.003	-0.003	-0.004	0.002	0.010	0.006	-0.004
153	NC_007384	Shigella sonnei Ss046	Proteobacteria; γ-	18	4825265	3824640	1412007	51.01	80.32	0.092	0.025	0.034	0.023	0.072	0.037	0.055	0.017	-0.003	-0.002	-0.003	0.006	0.014	0.010	-0.004
154	NC_007712	Sodalis glossinidius str. 'morsitans'	Proteobacteria; γ-	18	4171146	1	2085574	54.70	50.85	0.199	0.028	0.028	0.027	0.054	0.012	0.033	0.021	-0.008	-0.009	-0.007	-0.004	0.013	0.005	-0.008
155	NC_003143	Yersinia pestis CO92	Proteobacteria; γ-	18	4653728	4619729	2292865	47.64	80.17	0.097	0.030	0.036	0.029	0.083	0.038	0.061	0.023	-0.013	-0.016	-0.012	-0.018	0.004	-0.007	-0.011
156	NC_006155	Yersinia pseudotuberculosis IP 32953	Proteobacteria; γ-	18	4744671	4710647	2338311	47.61	82.47	0.178	0.044	0.052	0.043	0.089	0.024	0.056	0.033	-0.019	-0.024	-0.017	-0.022	0.011	-0.005	-0.016
157	NC_002971	Coxiella burnetii RSA 493	Proteobacteria; γ-	18	1995281	140	997777	42.66	84.61	0.195	0.049	0.028	0.053	0.082	-0.010	0.036	0.046	-0.002	-0.003	-0.002	0.009	0.018	0.014	-0.005
158	NC_006369	Legionella pneumophila str. Lens	Proteobacteria; γ-	18	3345687	192	1673035	38.41	86.99	0.150	0.070	0.074	0.069	0.118	-0.002	0.058	0.060	-0.019	-0.023	-0.019	0.010	0.057	0.034	-0.024
159	NC_002977	Methylococcus capsulatus str. Bath	Proteobacteria; γ-	18	3304561	3221107	1568826	63.58	89.27	0.078	0.038	0.055	0.036	0.031	-0.040	-0.005	0.036	-0.029	-0.042	-0.027	-0.015	0.041	0.013	-0.028
160	NC_008260	Alcanivorax borkumensis SK2	Proteobacteria; γ-	18	3120143	1	1560072	54.73	87.46	0.189	0.092	0.104	0.091	0.113	-0.058	0.028	0.086	-0.058	-0.060	-0.057	-0.052	0.064	0.006	-0.058
161	NC_007645	Hahella chejuensis KCTC 2396	Proteobacteria; γ-	18	7215267	378	3608011	53.87	87.85	0.124	0.059	0.074	0.058	0.087	-0.020	0.034	0.054	-0.022	-0.025	-0.021	0.004	0.055	0.030	-0.025
162	NC_002940	Haemophilus ducreyi 35000HP	Proteobacteria; γ-	18	1698955	1	849478	38.22	84.81	0.105	0.047	0.070	0.043	0.112	0.043	0.078	0.035	-0.005	0.010	-0.008	0.023	0.046	0.034	-0.011
163	NC_002663	Pasteurella multocida subsp. multocida str. Pm70	Proteobacteria; γ-	18	2257487	1000	1129743	40.40	88.95	0.019	0.007	0.008	0.007	0.083	0.072	0.077	0.006	-0.000	-0.002	0.000	0.019	0.019	0.019	-0.000
164	NC_005966	Acinetobacter sp. ADP1	Proteobacteria; γ-	18	3598621	201	1799511	40.43	87.82	0.238	0.057	0.091	0.054	0.099	0.019	0.059	0.040	-0.001	0.018	-0.004	-0.005	0.003	-0.001	-0.004
165	NC_007204	Psychrobacter arcticus 273-4	Proteobacteria; γ-	18	2650701	520	1325870	42.80	80.62	0.169	0.039	0.051	0.037	0.087	0.034	0.060	0.026	-0.000	0.004	-0.001	0.011	0.018	0.014	-0.004
166	NC_002516	Pseudomonas aeruginosa PA01	Proteobacteria; γ-	18	6264403	483	3132684	66.56	89.23	0.110	0.022	0.044	0.020	-0.010	-0.057	-0.034	0.023	-0.016	-0.036	-0.013	0.012	0.045	0.028	-0.017
167	NC_008027	Pseudomonas entomophila L48	Proteobacteria; γ-	18	5888780	552	2944942	64.16	88.72	0.145	0.032	0.049	0.030	0.010	-0.056	-0.023	0.033	-0.020	-0.034	-0.018	0.009	0.056	0.032	-0.023
168	NC_002947	Pseudomonas putida KT2440	Proteobacteria; γ-	18	6181863	9542	3100473	61.52	86.58	0.076	0.026	0.040	0.024	0.019	-0.031	-0.006	0.025	-0.020	-0.024	-0.019	0.001	0.042	0.022	-0.021
169	NC_004578	Pseudomonas syringae pv. tomato str.	Proteobacteria; γ-	18	6397126	339	3198902	58.40	85.07	0.065	0.024	0.039	0.022	0.030	-0.014	0.008	0.022	-0.024	-0.033	-0.022	-0.008	0.038	0.015	-0.023
170	NC_008245	Francisella tularensis subsp. tularensis FSC 198	Proteobacteria; γ-	18	1892616	1	946309	32.26	79.09	0.256	0.095	0.090	0.096	0.180	0.049	0.115	0.065	-0.003	-0.001	-0.003	0.034	0.066	0.050	-0.016
171	NC_006370	Photobacterium profundum SS9 # 1	Proteobacteria; γ-	18	4085304	7364	2050016	41.97	82.13	0.144	0.067	0.091	0.063	0.117	0.010	0.064	0.054	-0.023	-0.027	-0.022	-0.009	0.041	0.016	-0.025
172	NC_002505	Vibrio cholerae O1 biovar eltor str. N16961 # I	Proteobacteria; γ-	18	2961149	7397	1487971	47.69	87.52	0.206	0.053	0.068	0.051	0.084	-0.001	0.041	0.042	-0.012	-0.015	-0.011	-0.002	0.024	0.011	-0.013
173	NC_006840	Vibrio fischeri ES114 # I	Proteobacteria; γ-	18	2906179	7401	1460490	38.96	86.17	0.215	0.078	0.110	0.073	0.128	0.012	0.070	0.058	-0.009	-0.008	-0.009	0.008	0.037	0.022	-0.014
174	NC_004603	Vibrio parahaemolyticus RIMD 2210633 # I	Proteobacteria; γ-	18	3288558	7680	1651959	45.39	86.64	0.207	0.056	0.084	0.053	0.084	-0.006	0.039	0.045	-0.011	-0.020	-0.010	0.021	0.057	0.039	-0.018
175	NC_004459	Vibrio vulnificus CMCP6 # I	Proteobacteria; γ-	18	3281945	1005210	2646182	46.45	84.08	0.213	0.062	0.095	0.057	0.089	-0.008	0.040	0.048	-0.015	-0.038	-0.010	0.011	0.043	0.027	-0.016
176	NC_003919	Xanthomonas axonopodis pv. citri str. 306	Proteobacteria; γ-	18	5175554	42	2587819	64.77	85.85	0.128	0.022	0.029	0.020	0.027	-0.012	0.008	0.019	-0.015	-0.021	-0.014	-0.008	0.022	0.007	-0.015
177	NC_003902	Xanthomonas campestris pv. campestris str.	Proteobacteria; γ-	18	5076188	42	2538136	65.07	84.55	0.128	0.022	0.026	0.021	0.027	-0.013	0.007	0.020	-0.014	-0.015	-0.013	-0.006	0.023	0.008	-0.015
178	NC_006834	Xanthomonas oryzae pv. oryzae KACC10331	Proteobacteria; γ-	18	4941439	42	2470761	63.69	79.67	0.110	0.020	0.023	0.019	0.031	-0.004	0.013	0.018	-0.014	-0.015	-0.014	-0.004	0.026	0.011	-0.015
179	NC_002488	Xylella fastidiosa 9a5c	Proteobacteria; γ-	18	2679306	143	1339796	52.67	83.17	0.199	0.067	0.092	0.062	0.078	-0.039	0.019	0.058	-0.075	-0.091	-0.071	-0.079	0.059	-0.010	-0.069
180	NC_004342	Leptospira interrogans serovar Lai # I	Spirochaetes	19	4332241	234	2166354	35.01	77.89	0.172	0.068	0.068	0.068	0.116	0.000	0.058	0.058	-0.016	-0.022	-0.014	0.020	0.061	0.040	-0.020
181	NC_008277	Borrelia afzelii PKo	Spirochaetes	19	905394	460469	1	28.31	93.35	0.319	0.189	0.250	0.185	0.268	-0.013	0.128	0.141	-0.090	-0.114	-0.089	-0.025	0.208	0.092	-0.117
182	NC_001318	Borrelia burgdorferi B31	Spirochaetes	19	910724	456576	1	28.59	93.49	0.319	0.181	0.222	0.179	0.261	-0.010	0.126	0.135	-0.091	-0.113	-0.090	-0.025	0.211	0.093	-0.118
183	NC_006156	Borrelia garinii Pbi	Spirochaetes	19	904246	458789	1	28.30	92.73	0.324	0.177	0.241	0.173	0.257	0.001	0.129	0.128	-0.089	-0.114	-0.087	-0.022	0.210	0.094	-0.116
184	NC_002967	Treponema denticola ATCC 35405	Spirochaetes	19	2843201	321000	1742600	37.87	91.50	0.211	0.041	0.066	0.039	0.096	0.048	0.072	0.024	-0.010	-0.001	-0.010	0.052	0.107	0.079	-0.027
185	NC_000919	Treponema pallidum subsp. pallidum str. Nichols	Spirochaetes	19	1138011	4	569009	52.77	92.26	0.291	0.108	0.137	0.106	0.153	-0.023	0.065	0.088	-0.064	-0.061	-0.064	-0.097	0.001	-0.048	-0.049
